# Large language model analysis of real-world phone calls reveals prodromal and progressive biomarker of parkinsonism: A two-year proof-of-concept study

**DOI:** 10.1371/journal.pdig.0001458

**Published:** 2026-07-09

**Authors:** Martin Šubert, Vojtěch Illner, Michal Novotný, Tomáš Kouba, Tereza Tykalová, Michal Šimek, Pavel Sovka, Jan Švihlík, Evžen Růžička, Karel Šonka, Petr Dušek, Jan Rusz

**Affiliations:** 1 Department of Circuit Theory, Faculty of Electrical Engineering, Czech Technical University in Prague, Prague, Czech Republic; 2 Faculty of Chemical Engineering, University of Chemistry and Technology, Prague, Czech Republic; 3 Department of Neurology and Centre of Clinical Neuroscience, First Faculty of Medicine, Charles University and General University Hospital, Prague, Czech Republic; QIMR Berghofer: QIMR Berghofer Medical Research Institute, AUSTRALIA

## Abstract

No sufficiently sensitive biomarker exists to monitor disease progression to assess treatment efficacy in synucleinopathies such as Parkinson’s disease (PD), particularly during the prodromal phase when interventions are likely to be most effective. Existing digital biomarkers often rely on active tasks or clinic-based assessments, limiting their scalability and real-world applicability. In this proof-of-concept study, we evaluated whether linguistic features derived from real-world phone call recordings using large language models can serve as a language-based progression biomarker in isolated rapid eye movement sleep behavior (iRBD). In this two-year study, we enrolled 74 participants, including 21 iRBD (20 men), 26 PD (25 men), and 27 healthy controls (26 men) age-matched to iRBD participants. Speech data collection occurred remotely in participants’ natural environments through routine phone calls. Over 34,000 phone calls (<1,400 hours) were recorded over the two-year period. Compared to healthy controls, individuals with iRBD exhibited significant declines in sentence coherence (*p* = 0.016), semantic-syntactic diversity (*p* < 0.001), topic diversity (*p* < 0.001), and sentence probability (*p* < 0.001) over the two years. Prodromal changes in iRBD were detectable with an area under the curve of 0.82 from as few as 21 calls. For a two-year neuroprotective trial targeting 50% drug efficacy, the estimated sample size was 78 iRBD participants per arm based on a time-to-event analysis. These findings demonstrate that fully automated phone call analysis can detect both prodromal and progressive changes in alpha-synucleinopathy. This approach is scalable, minimizes the effort required from both patients and clinical staff, and enables remote, low-burden monitoring in screening at-risk populations and therapeutic trials.

## Author summary

Parkinson’s disease (PD) and related disorders begin years before clear clinical symptoms appear, creating a critical window for early intervention. However, there is still no sensitive and practical biomarker that can continuously monitor disease progression in daily life, especially during these early stages. In this study, we investigated whether natural speech collected during routine smartphone phone calls could provide such a marker. We developed a fully automated system that records and analyzes speech using artificial intelligence models designed to detect subtle changes in language patterns. Over two years, we collected more than 34,000 real-world phone calls from people with isolated rapid eye movement sleep behavior disorder, early PD, and healthy controls. We found that individuals at risk of developing PD showed progressive declines in several aspects of language, including sentence organization, vocabulary diversity, and conversational variability. These changes could be detected using as few as 21 phone calls and were more sensitive than standard laboratory speech tasks. Our findings suggest that passive analysis of everyday phone conversations may offer a scalable, low-burden approach for monitoring neurodegenerative disease progression and evaluating future disease-modifying treatments.

## Introduction

In alpha-synucleinopathies such as Parkinson’s disease (PD), identifying a reliable biomarker to assess the effectiveness of experimental treatments in slowing disease progression would represent a game-changing breakthrough [1,2]. The ideal biomarker should detect subtle changes even in the prodromal phase, the optimal window for introducing disease-modifying therapies [3–5]. Recent advances in digital health offer promising tools, such as smartphones, for remote, frequent, and non-invasive monitoring of early signs of parkinsonism and cognitive impairment [6,7]. However, most current digital approaches rely on active, guided tasks, such as finger tapping, walking a set distance or performing a cognitive test, and are validated only using cross-sectional data [6–8].

In contrast, a truly scalable digital biomarker would be captured passively during daily life without requiring additional effort from the individual or the clinician. In this context, speech analysis presents compelling advantages, as we use smartphones for daily communication. Speech reflects both cognitive-linguistic and motor-execution performance and is thus highly susceptible to neurodegeneration, providing a window into brain health [9]. Indeed, during office visits, experienced clinicians subconsciously recognize that PD is present, often only seconds after beginning a conversation with a patient [10]. However, existing speech-based digital biomarkers have mostly been validated in cross-sectional designs [11,12]. Currently, no language biomarkers have demonstrated the sensitivity required to reliably track parkinsonism progression and support their inclusion in future neuroprotective trials.

We present a fully automated smartphone-based system for passive and continuous monitoring of patients during routine phone calls in real-world conditions. Our approach leverages artificial intelligence, including large language models (LLMs) and sentence embeddings, to identify natural changes in language patterns associated with alpha-synucleinopathy, expressed through interpretable linguistic features. By assessing its ability to detect disease progression over two years, we validate this system in individuals with isolated rapid eye movement sleep behavior disorder (iRBD), a special case of prodromal alpha-synucleinopathy with high conversion rate to PD and dementia with Lewy bodies [1,3], compared to healthy controls and early-stage PD patients.

## Materials and methods

### Standard protocol approvals, registrations, and patient consents

The study was approved by the Ethics Committee of the General University Hospital in Prague, Czech Republic, and has been performed in accordance with the ethical standards laid down in the 1964 Declaration of Helsinki and its later amendments. All participants provided written, informed consent prior to their inclusion.

### Study design and participants

Between 2021 and 2022, Czech native participants were enrolled. Patients with iRBD were diagnosed based on polysomnography, according to the diagnostic criteria of the third edition of the International Classification of Sleep Disorders [13]. The exclusion criteria were as follows: (1) iRBD onset before age 50 years, (2) iRBD onset within 12 months of introduction of antidepressant treatment, (3) overt parkinsonism or dementia at baseline, (4) any therapy with antiparkinsonian medication, and (5) starting dopaminergic or cognitive therapy during 24 months of monitoring. Patients with PD were diagnosed using the Movement Disorder Society clinical diagnostic criteria for PD [14]. The exclusion criteria for PD were as follows: (1) disease duration from diagnosis >5 years, (2) not on a stable dose of medication over the previous 4 weeks prior to the start of the study, and (3) stopping medication during the study. The healthy control group was recruited to age- and sex-match the iRBD group. Exclusion criteria for healthy controls were a diagnosis of neurological disease or the diagnosis of RBD on video polysomnography. Exclusion criteria for all groups included (1) a history of communication disorders unrelated to parkinsonism (i.e., problems in speech comprehension or expression) or other neurological disorders potentially affecting speech, (2) involvement in any speech therapy, and (3) unwillingness to achieve at least 10 minutes of phone calls in a month.

### Laboratory assessment

The laboratory assessment was conducted at baseline and 24-month follow-up visit, and included testing of: (1) medical history including the use of medication; (2) motor symptoms using the Movement Disorders Society-Unified Parkinson’s Disease Rating Scale, Part III (MDS-UPDRS) [15]; (3) cognitive impairment using the Montreal Cognitive Assessment (MoCA) [16]; (4) autonomic symptoms using the Scales for Outcomes in Parkinson’s Disease–Autonomic Dysfunction (SCOPA-AUT) [17]; (5) Beck Depression Inventory-II (BDI-II) [18]; and (6) a two-minute monologue recorded during a face-to-face examination, in accordance with the guideline [19]. The estimation of symptom duration was based on the self-reported first occurrence of dream enactment in iRBD and motor symptoms in PD. The presence of mild cognitive impairment (MCI) was determined based on MoCA score lower than 1.5 standard deviation (SD) below the normative mean [16,20].

### Smartphone-based phone calls assessment

**Fig 1:**
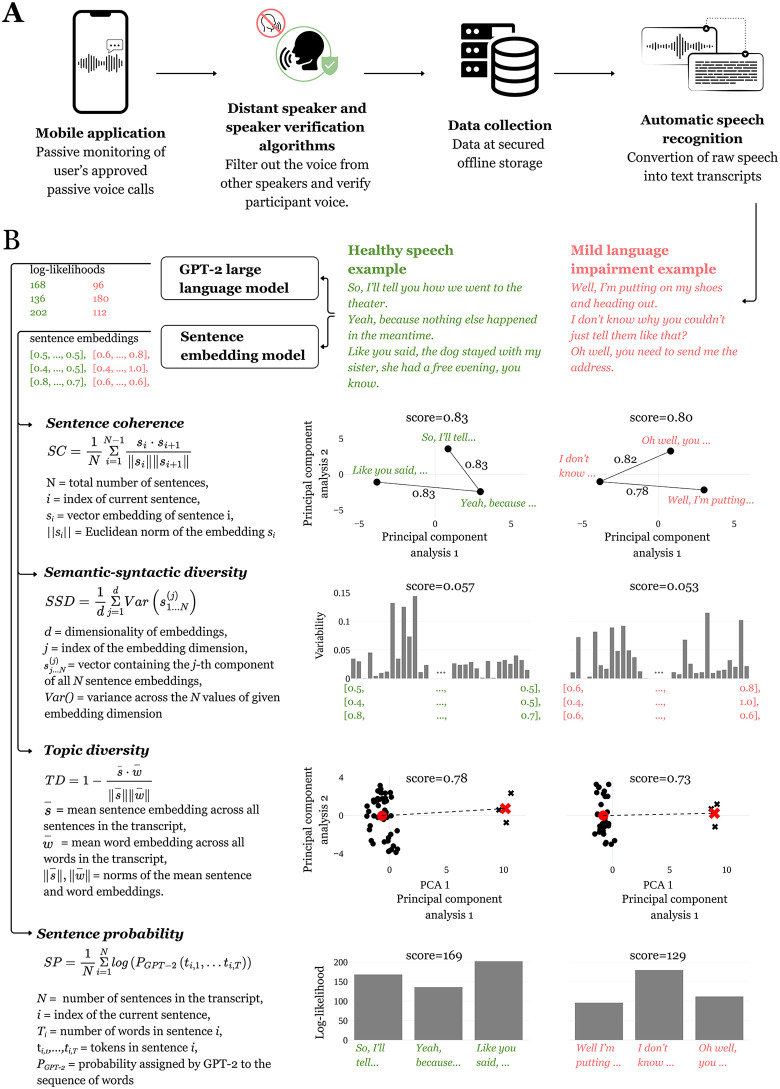
Diagram illustrating the phone call data collection and processing workflow for subsequent LLM-based linguistic analysis. **(A)** Data collection and pre-processing. Phone calls are passively recorded, filtered via speaker verification, securely stored, and transcribed using automatic speech recognition. **(B)** LLM-based linguistic modelling and feature extraction. Four features are derived: sentence coherence (SC), semantic-syntactic diversity (SSD), and topic diversity (TD) from sentence embeddings, and sentence probability (SP) from an autoregressive GPT-2 model. Colored columns compare healthy (green) and impaired (red) speech with corresponding feature scores.

Data were collected for up to 24 months following the in-person baseline clinic visit (**Fig 1A**), in compliance with the European Union’s personal data protection legislation. Each participant received an HONOR X9 Lite smartphone (Shenzhen Zhixin New Information Technology, Shenzhen, China) running Android 9, preinstalled with a custom-developed application [21] that recorded participants’ speech during phone calls. The application employed adaptive filtering to record only the participant’s speech. Recordings were sampled at 44.1 kHz, 16-bit, and stored locally for up to 24 hours, during which participants could review and delete them. If not deleted, recordings were pseudonymized and automatically uploaded to a secure server, where the speaker recognition system identifies the target participant within each recording. Audio recordings in which the participant was not detected (due to low audio quality, not able to identify human voice, or the participant not being the caller) were excluded from further analysis, serving as an initial quality control step. During the validation phase, a signal-to-noise estimator further excluded segments with an instantaneous signal-to-noise ratio below a predefined threshold. Comprehensive technical details of the application have been described in the protocol [21].

### Speech transcription

Each phone call recording was resampled to 16 kHz. Speech segments were detected using the pyannote voice activity detector [22]. Non-speech segments (silences) longer than five seconds were removed. Additionally, calls containing less than five seconds of participant speech were excluded from further analysis due to insufficient linguistic content. Recordings were transcribed using the Whisper automatic speech recognition system [23], employing the latest multilingual large-v3 model. To improve transcription accuracy and minimize hallucination errors (instances where the system inserts words not actually spoken, often due to background noise, speech irregularities, or model limitations), we developed a dedicated algorithm to detect and remove such artifacts. Specifically, phrases consisting of three or more words that are repeated consecutively at least four times were detected and removed. The selected parameters were defined and experimentally validated on an internal dataset comprising participants with iRBD and PD.

### Linguistic analysis

The selection of LLMs was based on open-source availability, full local executability, modest computational requirements, and reproducibility. All models were evaluated in a frozen-weight inference regime, without any task-specific fine-tuning. Transcription and linguistic analysis were performed entirely locally using offline models, with no data shared online. Two complementary approaches for linguistic analysis were adopted, using sentence embedding models and autoregressive LLMs (**Fig 1B**).

Sentence embedding models provide a numerical representation of sentences in a high-dimensional space, capturing semantic, syntactic, and contextual information within speech. We used RetroMAE-small, a sentence embedding model developed by Seznam.cz [24], selected for its compact 256-dimensional sentence representation and adapted on Czech data [24]. Each phone call transcript was processed in its entirety to preserve conversational meaning and contextual information. Each sentence was processed using the model’s tokenizer, which mapped words into numerical vectors (tokens). These tokens were then contextualized by the model to generate word embeddings that capture the surrounding linguistic context. We applied the max pooling method across the word embeddings and generated a final 256-dimensional sentence embedding vector. The max pooling method enhances key features and local variations within a sentence while minimizing the influence of irrelevant tokens, particularly beneficial in fragmented speech occurring in phone calls. We derived three one-dimensional linguistic features computed based on the sentence embeddings: (i) *sentence coherence*, defined as an average cosine similarity between consecutive sentence embeddings, reflecting the stability and consistency in semantic and syntactic speech in the conversation; (ii) *semantic-syntactic diversity*, quantified as the mean variance of sentence embeddings, capturing vocabulary richness, semantic expression and complexity of sentences; and (iii) *topic diversity*, computed as cosine distance between the average word embedding (red circle in **Fig 1B**) and the average sentence embedding (red cross in **Fig 1B**), reflecting thematic variability and descriptive creativity.

The second approach employed autoregressive LLM GPT-2 [25]. Although not fine-tuned particularly for Czech, to ensure full reproducibility, byte-level autoregressive modeling captures language-agnostic regularities in symbol sequencing and discourse structure [25, 26]. Using GPT-2, we derived (iv) *sentence probability,* defined as the average log-likelihood of each sentence under the GPT-2 model, where the model outputs the log-likelihood of the observed token sequence. This feature reflects the conformity to the conventional language patterns.

We also complemented LLM-based analyses with four state-of-the-art linguistic features based on natural language processing methods, including (i) *content richness*, (ii) *vocabulary range*, (iii) *sentence length*, (iv) *syntactic complexity* (**S1 Table**) [27].

### Statistical analysis

The primary outcome was the longitudinal change in linguistic characteristics over a 24-month period. We used linear mixed-effects models with the interaction between time and group (iRBD vs. controls and PD vs. controls) to evaluate whether patient groups exhibited faster progression than controls. Linear mixed-effects models handle unbalanced data caused by varying phone call counts and irregular timing. A participant-specific random effect accounted for within-subject correlations. Analysis was adjusted for age and education at baseline. Multiple comparisons across four LLM-based/standard linguistic features were adjusted using Bonferroni correction (<0.0125). Additionally, all LLM-based linguistic features were z-standardized and aggregated into a single *linguistic index*, defined as the mean of the standardized features. Beyond this, all statistical analyses were conducted on unnormalized, unstandardized features to ensure greater reproducibility.

As a secondary outcome, to assess the sensitivity of language analysis for potential screening, we assessed test-retest reliability using the intraclass correlation coefficient (ICC) to estimate the minimum number of calls required for consistent measurement, based on phone calls within 3 months of the initial visit. The reliability threshold was defined as the average ICC, and its 95% confidence interval exceeded 0.80. Baseline group differences were analyzed using analysis of covariance, adjusting for age and education. A binary logistic regression with leave-one-subject-out cross validation was used for baseline sensitivity analysis, reported as area under the curve (AUC), with features selected via an exhaustive search algorithm.

Furthermore, to assess the potential negative impact of the small number of females participants, we conducted sensitivity analysis including only male participants. To illustrate the potential of using linguistic biomarkers in clinical trials, we performed exploratory sample size calculation for 24-month neuroprotective trials assuming 50% drug efficacy. Sample sizes were estimated using the GLIMMPSE tool [28] for general linear mixed model power analysis; the Hotelling–Lawley Trace test was used with a target power of 0.80 and α = 0.05. Additionally, we performed a time-to-event analysis for a significant decline in linguistic features based on minimal detectable change, defined as the expected rate of change (slope) plus standard error in the healthy control group, estimated via linear regression. These estimates are intended to demonstrate the feasibility of linguistic biomarkers and are not prescriptive for clinical trial design. All statistical analyses were conducted using MATLAB R2024b, Python version 3.11, and GLIMMPSE tool.

## Results

### Participants

A total of 77 subjects, including 26 patients with PD, 24 subjects with iRBD, and 27 healthy controls, participated in the study; 3 iRBD subjects were subsequently excluded, 2 due to initiation of antiparkinsonian therapy and 1 due to initiation of cognitive therapy during the observed 24-month interval. Over the 2-year follow-up, MCI developed in two iRBD patients and two patients with PD (**Table 1**).

**Table 1:** Demographic and clinical data.

	Controls (n = 27)		iRBD (n=21)		PD (n = 26)		*p*-value
	*Baseline*	*24-month follow-up*	*Baseline*	*24-month follow-up*	*Baseline*	*24-month follow-up*	
Men	26 (96%)	–	20 (95%)	–	25 (96%)	–	1.0
Age (y)	67.5 (7.4)	–	67.9 (8.8)	–	57.9 (8.3)	–	<0.001^b,c^
Education (y)	17.5 (3.0)	–	15.3 (3.4)	–	15.6 (2.7)	–	0.025^a,b^
Self-reported symptom duration (y)	–	–	9.2 (6.5)	–	4.9 (2.3)	–	–
Disease duration (y)	–	–	3.7 (3.0)	–	2.7 (1.7)	–	–
MDS-UPDRS III, total	6.8 (2.7)	7.4 (4.8)	9.2 (3.2)^*^	11.3 (5.5)^*^	24.9 (8.8)	24.2 (9.2)	<0.001^b,c^
MDS-UPDRS III, speech item^€^	0.23 (0.43)^**^	0.56 (0.51)^**^	0.48 (0.51)^*^	0.81 (0.40)^*^	0.62 (0.34)^*^	1.16 (0.37)^*^	<0.001^b,c^
MoCA	25.8 (2.4)	26.7 (2.4)	26.7 (1.6)	26.1 (1.6)	27.2 (2.4)	27.2 (2.9)	0.13
MCI	0	0	0	2 (10%)	0	2 (8%)	1.0
SCOPA-AUT	7.6 (5.1)	7.5 (3.7)	12.8 (8.7)	13.9 (9.9)	8.7 (4.4)^*^	10.7 (5.3)^*^	0.015^a,c^
BDI II	5.1 (4.5)	5.0 (4.5)	8.3 (7.2)	7.9 (7.3)	6.0 (3.9)	6.2 (4.2)	0.11
Antidepressant therapy	1 (4%)	2 (7%)	2 (10%)	3 (14%)	4 (15%)	4 (15%)	0.37
Cognitive therapy	0	0	0	0	0	1 (4%)	1.0
Levodopa equivalent (mg/day)	0	0	0	0	523 (288)^***^	946 (485)^***^	<0.001^b,c^
Clonazepam therapy	0	0	8 (38%)	10 (48%)	1 (4%)	0	<0.001^a,c^
RBD presence	0	0	21 (100%)	21 (100%)	9 (35%)	9 (35%)	<0.001^a,b,c^

Data are presented as mean (standard deviation) or as number (%), as appropriate. Group differences at baseline were assessed using analysis of variance or the Kruskal–Wallis test and Dunn’s post-hoc test for continuous variables, and the Fisher’s exact tests for categorical variables. Significant changes between baseline and 24-month follow-up within each group were analyzed using the paired t-test or Wilcoxon signed-rank test for continuous variables, and McNemar’s test for categorical variables, and are indicated as * *p* < 0.05, ** *p* < 0.01, *** *p* < 0.001.

iRBD = isolated rapid eye movement sleep behavior disorder; PD = Parkinson’s disease; MDS-UPDRS = Movement Disorders Society-Unified Parkinson’s Disease Rating Scale; MoCA = Montreal Cognitive Assessment; MCI = mild cognitive impairment; SCOPA-AUT = Scales for Outcomes in Parkinson’s Disease-Autonomic Dysfunction; BDI = Beck Depression Inventory.

^a^Significant difference between healthy controls and iRBD.

^b^Significant difference between healthy controls and PD.

^c^Significant difference between iRBD and PD.

€ Perceptual speech severity was estimated using the speech item score from the MDS-UPDRS, Part III.

Over the 24 months, 34070 phone calls were collected. Of these, 33029 phone calls (mean 446, standard deviation [SD] 597 per participant) included more than 5 seconds of participant speech and were included in the analysis. The dataset included 1403 hours of participants’ speech (mean 19, SD 25 hours per participant). On average, each phone call included 2.6 (SD 3.2) minutes of participants’ speech (**S2 Table**). On a MacBook Air M1 with 16 GB RAM, processing a one-minute audio recording, from raw file to extracted linguistic features, takes approximately 70 seconds (SD 32), with 95% of the time spent on transcription and 5% on LLM-based modelling and feature extraction.

### Longitudinal changes in linguistic characteristics

**Fig 2:**
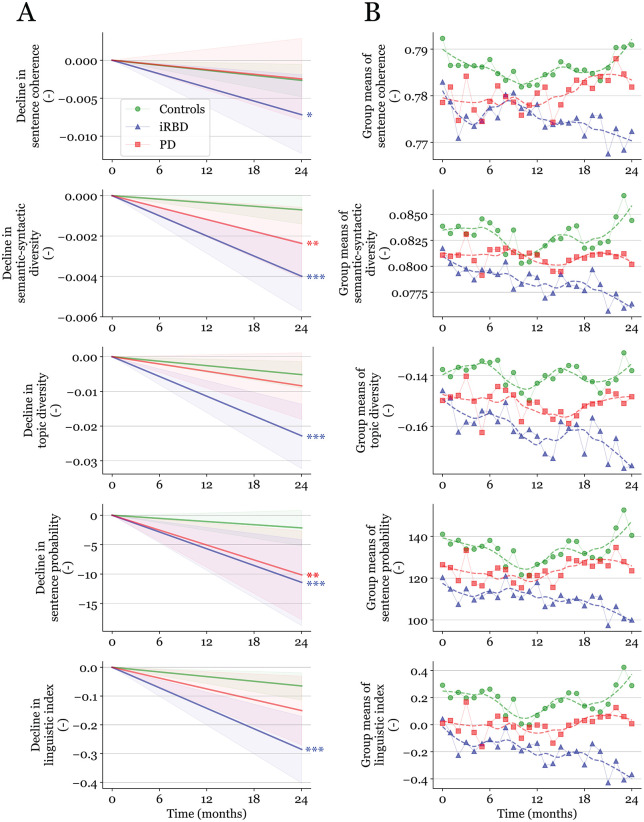
Longitudinal changes in LLM-based linguistic characteristics over 24 months based on real-world phone calls. **(A)** Group trajectories of linguistic change over the 24-month period. Each line represents estimated trajectory based on slopes derived from linear mixed-effects models, with shaded regions indicating the 95% confidence intervals. **(B)** Unadjusted monthly group-level averages of linguistic characteristics; these illustrative averages do not account for the varying number of phone calls across time points. Circles/triangles/squares represent the monthly means, and dashed lines depict smoothed trends calculated using the Savitzky–Golay filter. Statistical significance of group-by-time interaction (decline in iRBD vs. controls and PD vs. controls) based on linear mixed-effect models are indicated as follows: ^*^p < 0.05, ^**^p < 0.01, ^***^p < 0.001. iRBD = isolated rapid eye movement sleep behavior disorder; PD = Parkinson’s disease; LLM = large language model.

In the LLM-based linguistic analysis of phone calls, iRBD showed significant decline compared to controls in sentence coherence (β = -0.00019*, p* = 0.016), semantic-syntactic diversity (β = -0.00014*, p* < 0.001), topic diversity (β = -0.00074*, p* < 0.001), sentence probability (β = -0.39*, p* < 0.001), and linguistic index (β = -0.0092*, p* < 0.001) (**Fig 2**, **S3 Table**). The PD group demonstrated decline compared to controls in semantic-syntactic diversity (β = -0.0007*, p* = 0.008), and sentence probability (β = -0.33*, p* = 0.004). None of the covariates including age and education were significant for any linguistic characteristic. In an exploratory analysis, the two iRBD patients who developed MCI showed a significantly greater decline than iRBD without MCI in semantic-syntactic diversity (β = -0.00018, p = 0.012), sentence probability (β = -1.27, p < 0.001), and linguistic index (β = -0.014, p = 0.005) (**S1 Fig**); no significant change was observed for PD patients without MCI compared to PD patients with MCI. The standard linguistic analysis of phone calls revealed a decline in iRBD for content richness (β = -0.0037*, p* = 0.048) and PD in vocabulary range (β = -0.00065*, p* < 0.001) compared to controls (**S2 Fig**). Analysis of laboratory monologues showed no significant decline (**S3 Fig**). There were no significant correlations observed between change in language features and clinical scales.

### Sensitivity analysis for screening

**Fig 3:**
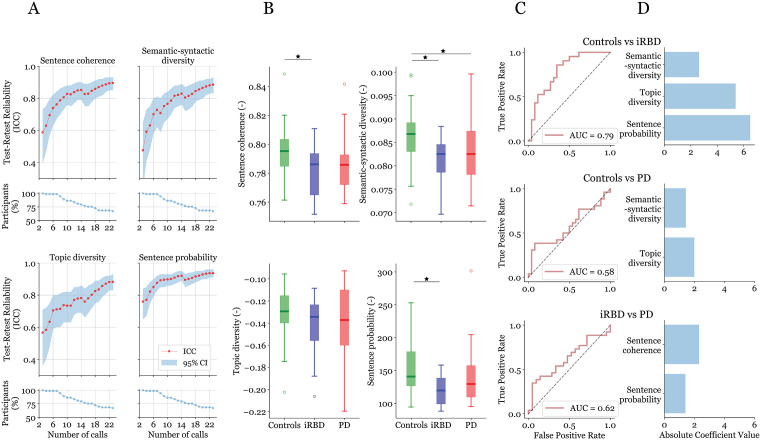
LLM-based linguistic analysis for screening derived from real-world phone calls within 3 months of initial visit. **(A)** Test-retest reliability of each LLM-based linguistic characteristic reported as intraclass correlation coefficients. **(B)** Group differences between individual LLM-based linguistic characteristics. The horizontal line represents the median, boxes represent the interquartile range (IQR), whiskers denote the minimum and maximum values within 1.5 × IQR; and circles indicate outliers. Significant group differences are marked as ^*^p < 0.05. **(C)** Receiver operating characteristic curves for binary logistic regression models with leave-one-subject-out cross validation using selected LLM-based linguistic characteristics. **(D)** Optimal LLM-based linguistic characteristics identified via an exhaustive search algorithm, along with their feature importances represented by the absolute coefficient value. ICC = intraclass correlation coefficient; CI = confidence interval; iRBD = isolated rapid eye movement sleep behavior disorder; PD = Parkinson’s disease; AUC = area under the curve; LLM = large language model.

The reliability threshold was achieved at 18 calls for sentence coherence, 20 for semantic-syntactic diversity, 21 for topic diversity, and 7 for sentence probability. This led to 21 calls per participant for baseline analyses to ensure reliability across all LLM-derived features (**Fig 3A**). The iRBD group differed significantly from controls in sentence coherence (*p* = 0.040), semantic-syntactic diversity (*p* = 0.029), and sentence probability (*p* = 0.012). PD differed from controls in semantic-syntactic diversity (*p* = 0.048) (**Fig 3B**). The highest classification performance was achieved between iRBD and healthy controls with an AUC of 0.79, whereas AUCs were 0.58 between PD and controls and 0.62 between iRBD and PD (**Fig 3C**, **Fig 3D**). Including only male participants, we achieved an AUC of 0.80 between iRBD and controls, 0.63 between PD and controls, and 0.58 between iRBD and PD (**S4 Fig**). Standard linguistic features (**S5 Fig**) and laboratory-based monologues (**S6 Fig**) demonstrated lower sensitivity for screening.

### Sample size estimation for clinical trials

For a 2-year neuroprotective trial targeting 50% drug efficacy, the estimated sample size was 180 participants per arm in iRBD for topic diversity and 470 in PD for semantic-syntactic diversity. The most efficient design employed time-to-event analysis, estimating 78 participants per arm in iRBD for sentence probability (**Table 2**).

**Table 2:** Estimated sample sizes for 24-month neuroprotective clinical trials at 50% drug efficacy.

Linguistic feature	Baseline	24-month prediction	Within-subject consistency	Residual standard deviation	Sample size per arm
*iRBD*					
Sentence coherence	0.794	0.787	0.07	0.032	1108
Semantic-syntactic diversity	0.078	0.074	0.39	0.010	262
Topic diversity	-0.166	-0.185	0.58	0.050	180
Sentence probability	137.74	127.14	0.74	43.56	278
Linguistic index	0.0375	-0.134	0.56	0.72	489
*iRBD, time-to-event analysis*					
Sentence coherence	0.802	0.788	0.07	0.031	311
Semantic-syntactic diversity	0.084	0.078	0.34	0.010	117
Topic diversity	-0.136	-0.165	0.52	0.049	88
Sentence probability	161.38	141.22	0.75	44.13	78
Linguistic index	0.449	0.182	0.54	0.72	212
*PD*					
Sentence coherence	0.794	0.791	0.22	0.032	7182
Semantic-syntactic diversity	0.078	0.076	0.62	0.010	470
Topic diversity	-0.166	-0.174	0.66	0.050	959
Sentence probability	144.55	134.33	0.58	43.56	479
Linguistic index	0.0801	-0.0284	0.72	0.72	777

Sample size estimates for a two-arm parallel trial (1:1 allocation), assuming 80% power and two-sided alpha of 0.05. Baseline values were estimated as the linear mixed-effects model intercepts, and 24-month values were calculated from monthly rate of change. Within-subject consistency was computed as the repeated measures correlation of feature over 24-month for each group. Residual standard deviations denote linear mixed-effects model residual variability. Time-to-event analysis for a significant linguistic decline was based on minimal detectable change, defined as the expected rate of change (slope) plus the standard error in the healthy control group, estimated via linear regression.

iRBD = isolated rapid eye movement sleep behavior disorder; PD = Parkinson’s disease.

## Discussion

This proof-of-concept study introduces a novel language-based progression biomarker for alpha-synucleinopathies effective from prodromal stages of disease that is derived from routine real-world phone calls and thus minimizes the effort required from both patients and clinical staff. Language represents a higher-order cognitive function encompassing grammar, vocabulary, syntax, and lexical processing, and is considered a strong predictor of cognitive impairment in a wide range of neurological conditions [29]. Yet, the production of spontaneous discourse in movement disorders might also be influenced by the impaired motor ability to articulate speech and construct longer, more complex sentences [30]. For a 2-year neuroprotective trial targeting 50% drug efficacy, the most efficient sample size using sentence probability marker was estimated at 78 iRBD participants per arm based on time-to-event analysis. This represents a substantial improvement over all existing state-of-the-art approaches investigated in the largest trial so far, including a variety of motor, cognitive, special sensory and autonomic variables, that suggest at least 117 participants per arm for a combination of cognitive and motor endpoints [31]. These findings illustrate the potential efficiency of linguistic biomarkers in this proof-of-concept study and are not intended as definitive trial design parameters. Unlike a previous study [31], we did not restrict our iRBD cohort to individuals meeting the Movement Disorder Society research criteria for prodromal PD [32], and we excluded patients who phenoconverted during the study. These differences may further reduce the required sample size under similar trial conditions. The traditional MoCA scale for assessing cognitive dysfunction was not sensitive enough to capture cognitive decline over time in our iRBD cohort. Indeed, the two iRBD participants who developed MCI exhibited a steeper linguistic decline and may indicate a potential association between linguistic decline and emerging cognitive deterioration earlier than conventional screening tools. However, this finding should be interpreted with caution given the very small sub-sample. As we did not find any relationship between change in linguistic features and clinical scales, we may hypothesize language impairment to be a unique, non-overlapping marker of disease severity compared to gold-standard clinical scales. Notably, although both the MDS-UPDRS III total score and its speech item declined over time, neither was associated with changes in linguistic features. This dissociation suggests that the observed linguistic alterations predominantly reflect non-motor language processes rather than motor speech impairment. Moreover, speech assessment is inherently scalable and can be used to screen individuals at risk of developing overt synucleinopathies. In our study, only 21 phone calls were sufficient to detect prodromal linguistic changes, achieving a robust AUC of 0.82.

### Longitudinal linguistic changes derived from phone call speech

Over 24 months, we collected more than 34,000 phone call recordings in real-world conditions and demonstrated that LLM-based linguistic characteristics can indeed detect subliminal language decline in both iRBD and PD cohorts. iRBD is now recognized as a key prodromal indicator of alpha-synucleinopathies, with most individuals progressing to overt neurodegenerative disease, predominantly PD or dementia with Lewy bodies [1,33]. In this study, iRBD showed a more pronounced decline across all LLM-based linguistic features than the PD group. Several reasons may explain this finding. The more disturbed iRBD language profile can be influenced by increased proportion of prodromal dementia with Lewy bodies within this group [34]. The presence of RBD within PD represents a subtype with more akinetic-rigid disease, autonomic symptoms, and greater cognitive impairment [35,36], while our PD cohort was predominantly without RBD. PD patients were on stable dopaminergic therapy, which might ameliorate some language manifestations [27]. Finally, iRBD often manifests later in life compared to a naturalistic PD cohort [2,37], resulting in our early-stage PD group being, on average, 10 years younger than the iRBD and control groups. Although we did not observe a significant effect of age in our analyses, ageing might still have some effect on language performance [38], and thus supporting the better performance observed in our PD group.

### Linguistic abnormalities and its pathophysiology

From a pathophysiological perspective, iRBD exhibited a more pronounced decline in sentence coherence, a key indicator of discourse stability. This decline reflects the increased difficulty in maintaining logical flow, potentially indicating an early disruption in thought organization. Both iRBD and PD demonstrated reduction in semantic-syntactic diversity, indicating restricted vocabulary, which aligns with prior findings in iRBD [39]. Moreover, limited vocabulary range was also identified as a strong predictor of phenoconversion into defined alpha-synucleinopathies [9]. The presence of syntactic deficits further emphasized the simplicity of speech, characterized by repetitive, uniform patterns and reduced sentence complexity. Similar speech patterns were previously reported in iRBD patients with MCI [39]. Reduced topic diversity revealed a narrowing scope in conversational themes, using generic, simplistic expressions. Lastly, sentence probability was computed using a GPT-2 model trained on approximately 8 million web pages [25], emphasizing quality content representative of the natural language use. A progressive decline in this measure in both iRBD and PD suggests that patients’ speech gradually diverges from typical linguistic patterns observed in the general population.

### Advantages and impact of passive monitoring in naturalistic environment

Unlike traditional laboratory-based assessments, our passive monitoring approach in a natural environment eliminates confounding factors such as practice effect [40], anxiety, fatigue, and other visit-related stressors. Crucially, it allows high-frequency observation of subtle linguistic patterns often missed in structured tasks. The large volume of 34,000 phone calls, averaging approximately 19 calls per participant per month, enabled near-continuous insight into patient condition and fine-grained progression tracking. Indeed, phone calls considerably outperformed standard one-per-year laboratory monologues in every analysis. We demonstrated that, using a single mid-range MacBook, processing a one-minute audio recording takes slightly over one minute, mostly consumed by the ASR. We used the base Whisper large-v3 model, which is not optimized for computational efficiency, although alternative approaches can reduce processing time without sacrificing accuracy, such as *faster-whisper* [41]. Future optimization could enable smartphones to extract linguistic features directly on-device, eliminating the need for audio transfer and enhancing user privacy. Recent benchmark comparisons indicate that, on a mid-range smartphone with an optimized inference engine, one-minute audio can be transcribed in 7–41 seconds (real-time factor 0.07–0.41), whereas desktop systems achieve 3-20x faster transcription depending on model size and inference backend [42]. Moreover, the transferability of our approach likely extends beyond parkinsonism and alpha-synucleinopathies, as similar linguistic disruptions appear across other neurodegenerative disorders [43,44].

### LLM-based approach for linguistic analysis

Compared to standard linguistic analysis in controlled, laboratory-based tasks, the challenge of real-world communication is that it introduces intra-speaker variability, influenced by social context (talking to a relative versus a coworker) or communication goals (informative versus emotional). LLMs offer a high-dimensional, contextualized understanding of language that is more robust to this variability [45]. In our reliability analysis, LLM-derived characteristics required only 21 phone calls to achieve stable measurement across all four features. Most standard features failed to meet this threshold (**S5A Fig**), showing greater measurement instability. Additionally, LLM-based features showed greater sensitivity to language decline, even with fewer phone calls. Many state-of-the-art models are open-source and multilingual, making them ideal for cross-linguistic and cost-effective use. To avoid potential overfitting, we did not use present speech data to fine-tune deployed models. However, LLMs can be adapted to disease-specific speech to enhance future sensitivity to subtle progression markers and enable more personalized monitoring.

### Limitations

Potential limitations should be noted. This is a single-center, proof-of-concept study, where we had a limited opportunity to recruit more iRBD subjects due to its low prevalence, with approximately 50% of subjects willing to participate [46]. Using a single smartphone model ensured consistent audio quality in this proof-of-concept phase. Future studies should test across devices, though prior study shows minimal impact of microphone type on transcription accuracy [47]. The transcription errors arising from dysarthric speech and limitations of the automatic speech recognition system could potentially affect the analysis. However, a previous study shows that linguistic analysis remains robust to transcription errors, with strong correlations (r > 0.90) between manual and automated features even at word error rates up to 18.7% in iRBD and PD cohorts [39]. The study sample is predominantly males, with only one female participant in each group, which limits the generalizability of the findings to female patients. This imbalance reflects the naturally high male predominance in iRBD, which can be as high as 90% [48]. Our control group had, on average, two more years of education than the patient cohorts; however, education did not significantly influence the results. Despite using only Czech data, prior study has already shown that linguistic analysis in iRBD is language-independent [9].

## Conclusion

In this proof-of-concept study, LLM-based analysis of routine phone calls revealed natural linguistic abnormalities associated with prodromal and early-stage alpha-synucleinopathy, capturing their progressive decline over time. This passive, low-burden approach enables continuous monitoring in real-world settings, positioning linguistic features as a promising digital biomarker of alpha-synucleinopathy progression, with the potential to redefine clinical trial endpoints in future neuroprotective interventions. Validation in larger, multicenter cohorts is now essential. Future studies may benefit from combining LLM-based linguistic features with motor speech measures to further enhance predictive modeling.

## Supporting information

S1 Table Description of standard linguistic characteristics. (DOCX)

S2 Table Descriptive statistics of the total number and duration of phone calls. iRBD = isolated rapid eye movement sleep behavior disorder; PD = Parkinson’s disease.
(DOCX)

S3 Table Descriptive statistics of LLM-based linguistic features at baseline, 1-year, and 2-year follow-up. Data are presented as mean (standard deviation). iRBD = isolated rapid eye movement sleep behavior disorder; PD = Parkinson’s disease.
(DOCX)

S1 Fig Longitudinal changes of LLM-based linguistic characteristics over 24 months derived from real-world phone calls in iRBD with and without mild cognitive impairment. **(A)** Individual trajectories of linguistic change over the 24-month period. Each line represents estimated trajectory based on slopes derived from linear mixed-effects models, with shaded regions indicating the 95% confidence intervals. Statistical significance of group-by-time interaction (decline in iRBD with MCI vs. iRBD without MCI) based on linear mixed-effects models are indicated as follows: ^*^p < 0.05, ^**^p < 0.01, ^***^p < 0.001. **(B)** Monthly group-level averages of linguistic characteristics; these illustrative averages do not account for the varying number of phone calls across time points. Circles/triangles/squares represent the monthly means, and dashed lines depict smoothed trends calculated using the Savitzky–Golay filter. iRBD = isolated rapid eye movement sleep behavior disorder; MCI = mild cognitive impairment; LLM = large language model.
(EPS)

S2 Fig Longitudinal changes of standard linguistic characteristics over 24 months derived from real-world phone calls. **(A)** Individual trajectories of linguistic change over the 24-month period. Each line represents estimated trajectory based on slopes derived from linear mixed-effects models, with shaded regions indicating the 95% confidence intervals. Statistical significance of group-by-time interaction (decline in iRBD vs. controls and PD vs. controls) based on linear mixed-effect models are indicated as follows: ^*^p < 0.05, ^***^p < 0.001. **(B)** Monthly group-level averages of linguistic characteristics; these illustrative averages do not account for the varying number of phone calls across time points. Circles/triangles/squares represent the monthly means, and dashed lines depict smoothed trends calculated using the Savitzky–Golay filter. iRBD = isolated rapid eye movement sleep behavior disorder; PD = Parkinson’s disease; LLM = large language model.
(EPS)

S3 Fig Longitudinal changes in LLM-based linguistic characteristics derived from laboratory monologue task at baseline and at 24 months. Each participant was asked to engage in spontaneous discourse on a topic of their choice (monologue) with an average duration of 2.0 (SD 0.3) minutes. The monologue was recorded in a single session in a quiet room using a head-mounted condenser microphone (Beyerdynamic Opus 55, Heilbronn, Germany) positioned approximately 5 cm from the participant’s mouth. Recordings were made at a sampling rate of 48 kHz and 16-bit resolution. **(A)** Individual trajectories of linguistic change over the 24-month period. Each line represents estimated trajectory based on slopes derived from linear mixed-effects models, with shaded regions indicating the 95% confidence intervals. **(B)** Monthly group-level averages of linguistic characteristics. Circles/triangles/squares represent the monthly means. iRBD = isolated rapid eye movement sleep behavior disorder; PD = Parkinson’s disease; LLM = large language model.
(EPS)

S4 Fig Sensitivity analysis based on LLM-based linguistic features derived from real-world phone calls within 3 months of initial visit including only male participants. **(A)** Receiver operating characteristic curves for binary logistic regression models with leave-one-subject-out cross validation using selected linguistic characteristics. (**B)** Optimal linguistic characteristics identified via an exhaustive search algorithm, along with their feature importances represented by the absolute coefficient value. iRBD = isolated rapid eye movement sleep behavior disorder; PD = Parkinson’s disease; AUC = area under the curve.
(EPS)

S5 Fig Standard linguistic analysis for screening derived from real-world phone calls within 3 months of initial visit. **(A)** Test-retest reliability of each linguistic characteristic reported as intraclass correlation coefficients. **(B)** Group differences between individual linguistic characteristics. The horizontal line represents the median, boxes represent the interquartile range (IQR), whiskers denote the minimum and maximum values within 1.5 × IQR; and circles indicate outliers. Significant group differences are marked as ^*^p < 0.05. **(C)** Receiver operating characteristic curves for binary logistic regression models with leave-one-subject-out cross validation using selected linguistic characteristics. **(D)** Optimal linguistic characteristics identified via an exhaustive search algorithm, along with their feature importances represented by the absolute coefficient value. ICC = intraclass correlation coefficient; CI = confidence interval; iRBD = isolated rapid eye movement sleep behavior disorder; PD = Parkinson’s disease; AUC = area under the curve.
(EPS)

S6 Fig Baseline LLM-based linguistic analysis derived from two-minute laboratory monologues. Each participant was asked to engage in spontaneous discourse on a topic of their choice (monologue) with average duration of 2.0 (SD 0.3) minutes. The monologue was recorded in a single session within a quiet room using a head-mounted condenser microphone (Beyerdynamic Opus 55, Heilbronn, Germany) positioned approximately 5 cm from the participant’s mouth. Recordings were made at a sampling rate of 48 kHz and 16-bit resolution. **(A)** Group differences between individual linguistic characteristics. The horizontal line represents the median, boxes represent the interquartile range (IQR), whiskers denote the minimum and maximum values within 1.5 × IQR; and circles indicate outliers. **(B)** Receiver operating characteristic curves for binary logistic regression models with leave-one-subject-out cross validation using selected linguistic characteristics. **(C)** Optimal linguistic characteristics identified via an exhaustive search algorithm, along with their feature importances represented by the absolute coefficient value. ICC = intraclass correlation coefficient; CI = confidence interval; iRBD = isolated rapid eye movement sleep behavior disorder; PD = Parkinson’s disease; AUC = area under the curve.
(EPS)
