# Supplementary material for: Large language model analysis of real-world phone calls reveals prodromal and progressive biomarker of parkinsonism: A two-year proof-of-concept study
Source: PLOS Digit Health. 2026 Jul 9;5(7):e0001458. doi: 10.1371/journal.pdig.0001458 (PMC13349142; doi:10.1371/journal.pdig.0001458)
Supplement: S1 Table — (DOCX) [file pdig.0001458.s001.docx]

**S1 Table Description of standard linguistic characteristics.**

| Linguistic characteristic | Feature description | Reference |
| --- | --- | --- |
| Content richness | Measured using content density: the ratio of open-class (content) words to closed-class (function) words. | *Orimaye SO, Wong JS, Golden KJ, Wong CP, Soyiri IN. Predicting probable Alzheimer's disease using linguistic deficits and biomarkers. BMC Bioinformatics. 2017 Jan 14;18(1):34. doi: 10.1186/s12859-016-1456-0.* |
| Vocabulary range | Measured using moving-average type-token ratio: the average ratio of unique to total words across overlapping windows. Higher values indicate richer vocabulary. | *Covington MA, McFall JD. Cutting the Gordian knot: the moving- average type–token ratio (MATTR). J Quantit Ling 2010 May 14;17(2):94–100.  doi: 10.1080/09296171003643098.* |
| Sentence length | Measured using mean length of utterance: the average number of words per sentence. | *Subert M, Tykalova T, Novotny M, Bezdicek O, Dusek P, Rusz J. Long-term dopaminergic therapy improves spoken language in de-novo Parkinson's disease. J Neurol. 2025 Apr 17;272(5):344. doi: 10.1007/s00415-025-13070-8.* |
| Syntactic complexity | Measured using syntactic dependency length: the average distance between heads and dependents in syntactic dependency trees. | *Subert M, Tykalova T, Novotny M, Bezdicek O, Dusek P, Rusz J. Long-term dopaminergic therapy improves spoken language in de-novo Parkinson's disease. J Neurol. 2025 Apr 17;272(5):344. doi: 10.1007/s00415-025-13070-8.* |
