# Supplementary material for: Large language model analysis of real-world phone calls reveals prodromal and progressive biomarker of parkinsonism: A two-year proof-of-concept study
Source: PLOS Digit Health. 2026 Jul 9;5(7):e0001458. doi: 10.1371/journal.pdig.0001458 (PMC13349142; doi:10.1371/journal.pdig.0001458)
Supplement: S2 Table — iRBD = isolated rapid eye movement sleep behavior disorder; PD = Parkinson’s disease. (DOCX) [file pdig.0001458.s002.docx]

**S2 Table Descriptive statistics of the total number and duration of phone calls.**

|  | Group | Baseline  (0-2 months) | 1-year follow-up  (11-13 months) | 2-year follow-up  (22-24 months) |
| --- | --- | --- | --- | --- |
| Total number of phone calls | Controls  iRBD  PD | 1172  886  1090 | 1408  1174  992 | 1186  1388  1103 |
| Total speech duration (hours) | Controls  iRBD  PD | 54.6  46.4  48.6 | 58.2  45.1  41.1 | 53.4  46.5  40.9 |

iRBD=isolated rapid eye movement sleep behavior disorder; PD=Parkinson’s disease.
