# Supplementary material for: Large language model analysis of real-world phone calls reveals prodromal and progressive biomarker of parkinsonism: A two-year proof-of-concept study
Source: PLOS Digit Health. 2026 Jul 9;5(7):e0001458. doi: 10.1371/journal.pdig.0001458 (PMC13349142; doi:10.1371/journal.pdig.0001458)
Supplement: S3 Table — Data are presented as mean (standard deviation). iRBD = isolated rapid eye movement sleep behavior disorder; PD = Parkinson’s disease. (DOCX) [file pdig.0001458.s003.docx]

**S3 Table Descriptive statistics of LLM-based linguistic features at baseline, 1-year, and 2-year follow-up.**

| Linguistic characteristic | Group | Baseline  (0-2 months) | 1-year follow-up  (11-13 months) | 2-year follow-up  (22-24 months) |
| --- | --- | --- | --- | --- |
| Sentence coherence | Controls  iRBD  PD | 0.786 (0.033)  0.775 (0.033)  0.779 (0.035) | 0.783 (0.033)  0.776 (0.035)  0.780 (0.035) | 0.791 (0.033)  0.771 (0.033)  0.785 (0.037) |
| Semantic-syntactic diversity | Controls  iRBD  PD | 0.083 (0.011)  0.080 (0.011)  0.081 (0.012) | 0.081 (0.011)  0.079 (0.012)  0.081 (0.011) | 0.085 (0.011)  0.077 (0.012)  0.081 (0.012) |
| Topic diversity | Controls  iRBD  PD | -0.138 (0.045)  -0.157 (0.063)  -0.147 (0.061) | -0.144 (0.047)  -0.165 (0.070)  -0.152 (0.068) | -0.137 (0.052)  -0.172 (0.076)  -0.149 (0.059) |
| Sentence probability | Controls  iRBD  PD | 135.614 (58.591)  111.833 (43.471)  123.537 (48.614) | 125.413 (53.499)  113.325 (45.285)  122.060 (44.975) | 145.736 (58.473)  102.426 (41.215)  128.626 (53.802) |
| Linguistic index | Controls  iRBD  PD | 0.208 (0.764)  -0.152 (0.776)  0.022 (0.817) | 0.064 (0.764)  -0.186 (0.798)  -0.011 (0.782) | 0.336 (0.815)  -0.348 (0.807)  0.063 (0.816) |

Data are presented as mean (standard deviation).

iRBD=isolated rapid eye movement sleep behavior disorder; PD=Parkinson’s disease.
