# Supplementary material for: Large language model analysis of real-world phone calls reveals prodromal and progressive biomarker of parkinsonism: A two-year proof-of-concept study
Source: PLOS Digit Health. 2026 Jul 9;5(7):e0001458. doi: 10.1371/journal.pdig.0001458 (PMC13349142; doi:10.1371/journal.pdig.0001458)
Supplement: Attachment — Submitted filename: ResponseToReviews_R1.pdf [file pdig.0001458.s011.pdf]

Specific responses to reviewers' comments:

Reviewer #1:

This is an interesting and timely study that addresses the unmet need for early and sensitive biomarkers of synucleinopathies, a topic of substantial relevance to the field of neurodegenerative diseases. The use of LLM-based analysis of real-world telephone speech, combined with a longitudinal and remote design, represents a novel and scalable approach to disease monitoring. The manuscript is clearly written and well structured; however, several aspects require clarification and further substantiation.

1. The study sample is predominantly males, with only one female participant in each subgroup, which is not currently discussed or addressed in the manuscript.

a. Sex differences are known to influence both Parkinsonian manifestations and language characteristics. The inclusion of a single female participant per group introduces the potential for outlier effects, where a small number of atypical datapoints (due to sex-based differences) could disproportionately influence estimates or variance. A sensitivity analysis restricted to male participants would help assess the robustness of the reported findings.

- *Thank you for your comment. We conducted an additional sensitivity analysis including only male participants, which yielded very similar but slightly better results: the AUC for iRBD versus healthy controls was 0.79 (including females) versus 0.80 (males only); for PD versus controls, 0.58 versus 0.62; and for iRBD versus PD, 0.62 versus 0.58. The corresponding ROC curves and feature importances are provided in the supplement as Figure S3. This analysis was performed using leave-one-subject-out cross-validation, as recommended (see further response below).*

b. This extreme sex imbalance of sample composition limits the interpretability and generalizability of the findings to female patients, as. The authors should explicitly acknowledge this limitation in the Methods, Results, and Discussion sections, and consider mentioning it briefly in the Abstract.

- *We have added information about participant sex to the abstract. The Methods and Results sections now include the sensitivity analysis restricted to male participants. In the Discussion, under study limitations, we note that the sample is predominantly male, with only one female participant per group, which limits the generalizability of the findings to female patients. However, this imbalance reflects the naturally high male predominance in iRBD (i.e., our primary target population), which can reach up to 90%. Thus we could not influence this gender imbalance by better design of the study.*

2. This study used the Whisper large-v3 model for audio transcription.

a. While Whisper is highly accurate for general ASR, hallucination is a common issue due to its extensive training on sources such as YouTube. The model can fabricate words or sentences (e.g., “thanks for watching,” “thank you”, etc.) that were not actually spoken, particularly during periods of silence, and it can also repeat phrases or get stuck in loops. The authors mention applying an algorithm to detect and remove these artifacts, but no details were provided regarding how this was performed (automatic or manual, using existing tools or a self-developed method) and how effectively it removed such errors. This is a crucial point, as the linguistic measures of interest in this study are sentence-based.

- *Thank you for pointing this out. The algorithm was developed by our lab. It detects and removes phrases of at least three words in length that are repeated consecutively four or more times. These parameters were established and validated based on internal dataset involving participants with iRBD and PD, in which similar transcription artifact was also*

observed. This information has been added to the Methods section under Speech transcription. Due to privacy policy, we should not manually read or transcribe the phone call recordings, and therefore we did not calculated exact efficacy on these voice calls.

b. The study used a custom-developed application that recorded only the participant's speech during phone calls. Were trailing silences or long within-utterance pauses removed during audio processing of these recordings? This is important, as prolonged silences could affect Whisper's transcription accuracy and potentially introduce artifacts, as noted above.

- *We agree that this information is missing. We removed silences and non-speech segments in the audio that was longer than 5 seconds. Such removal should improve the transcription and increase the speed of transcription process. This information was also added to Methods section under Speech transcription.*

c. Transcription errors, especially in the context of dysarthric speech, and autocorrection could also bias the results. The authors should clarify whether these issues were checked and should at minimum acknowledge them as limitations.

- *Similarly as in the case of hallucinations, due to privacy policy, we should not manually read or transcribe the phone call recordings, making it impossible to precisely report the transcription accuracy using these voice calls. However, our previous study indicate that linguistic analysis is robust toward potential transcription error. The* study *(10.1002/mds.29140) compared linguistic features extracted based on automated versus manual transcripts in iRBD and Parkinson's disease, demonstrating high correlation between features (r>0.9) even when the word error rate was up to 18.7%. Nevertheless, errors arising from dysarthric speech or limitations of automatic speech recognition system could still affect the analysis, and we have acknowledged this in the Discussion as a study limitations.*

3. Could the authors provide more details on audio processing? Were recordings discarded only based on insufficient linguistic content (e.g., calls with <5 seconds of participant speech)? Were all retained recordings of sufficient quality for transcription and feature extraction? Were any recordings excluded for other reasons?

- *Once the audio recording is uploaded to our server, the speaker recognition system is designed to detect the target participant. Audio segments in which the participant is not detected—due to low audio quality, not able to identify human voice, or the participant not being the caller—are excluded from further analysis, serving as an initial quality control step. During the validation phase, a signal-to-noise estimator further excludes segments with an instantaneous signal-to-noise ratio below a predefined threshold. This pipeline was developed and tested to work also for acoustic analysis, where the demand for quality audio is significantly higher. A detailed description of the data acquisition process and particular algorithms have been previously published in the protocol (please, see https://doi.org/10.1136/bmjopen-2021-059871). Additionally, we employed the pyannote voice activity detector to identify voice activity and removed segments in which participants spoke for less than 5 seconds. We have substantially revised the Materials and methods section to provide a clearer introduction and description of the pipeline.*

4. The study reported relatively high accuracy; however:
a. Validation or test set evaluation are necessary to assess the model's generalizability and its applicability to real-world data. Given the study sample size, a conventional train/test split may

not be feasible. The authors could consider holding out a subset of phone calls, cross-validation, or, if possible, an external dataset to evaluate the robustness and generalizability of the model.

- *Thank you for your comment and suggested solutions. Given the available sample size, we implemented leave-one-subject-out cross-validation. All results for the sensitivity analysis are now based on binary logistic regression with leave-one-out cross-validation.*

b. The modelling was performed separately for each linguistic domain, which is appropriate for understanding specific language changes and identifying features most relevant to disease progression. Nonetheless, the authors could also consider a combined modelling approach across domains. This would provide a more comprehensive capture of language alterations and could enhance predictive performance.

- *Thank you for your suggestion. For the LLM-based features, we constructed a linguistic index defined as the mean of the z-standardized feature values. This index was incorporated into the longitudinal analysis (Figure 1) and the sample size estimation (Table 2).*

c. Although both the MDS-UPDRS III total and speech item declined over time, they showed no correlation with language changes. This suggests that the observed linguistic alterations reflect non-motor processes and that incorporating motor speech features could further enhance the model's predictive performance. While this may be beyond the scope of the current study, the authors could consider discussing this point, as it may have implications for future predictive modelling that fully leverages speech analysis and biomarkers.

- *We agree. We have expanded the Discussion section to explicitly address this finding and its implications. Specifically, we added: „*Notably, although both the MDS-UPDRS III total score and its speech item declined over time, neither was associated with changes in linguistic features. This dissociation suggests that the observed linguistic alterations predominantly reflect non-motor language processes rather than motor speech impairment.“

5. Finally, a minor point regarding the statistical tests used to compare demographic and clinical characteristics: several categorical variables have very small cell sizes (<5), making the Chi-squared test inappropriate. Fisher's exact test should be used instead for these comparisons.

- *Thank you for this comment. Upon review of our analysis, we confirmed that all categorical variables should be analyzed using Fisher's exact test. The analysis and corresponding results have been updated accordingly.*

6. A few minor typographical points:
a) In the legend of Figure 2, panel (D) reads "Optimal LLM-based linguistic characteristics identifies via …"; the authors likely meant "identified."
b) In Figure 3, the "sentence probability" section may contain a typo: "language patters" should probably be "language patterns."

- *Thank you, the typographical errors were corrected.*

Reviewer #2:
General Comments:
The authors presented an innovative and highly promising approach that leverages large language models (LLMs) to extract linguistic biomarkers (digital bio-markers) from real-world phone calls for monitoring disease progression in isolated REM sleep behavior disorder (iRBD) and Parkinson's disease (PD). The reported linguistic biomarker could possibly be used in future disease-modifying trials.

The study's design, using passive, naturalistic phone conversations, is novel and aligns with current trends in developing scalable, low-burden digital health biomarkers.
From the AI/LLM standpoint, the concept and rationale are strong, and the integration of sentence embeddings (RetroMAE-small) and GPT-2–derived linguistic metrics is appropriate for capturing cognitive-linguistic decline.

- *Thank you for your positive comments.*

Major Comments:

1. Due to the low sample size, I would highly recommend using the word "proof-of-concept" in the title & the main body of text, i.e., methods section, and also in the abstract.

- *We have incorporated the phrase "proof-of-concept" into the title and abstract. Additionally, we have used it several times throughout the main body of the manuscript to emphasize that this study represents a pilot proof-of-concept investigation, as further highlighted in our discussion of sample size considerations.*

2. Strength: The inclusion of a test–retest reliability analysis using ICC is commendable and demonstrates thoughtful attention to the intra-individual stability of the LLM-derived linguistic features. Establishing that 18–21 phone calls are sufficient for consistent measurement is a useful step toward defining the data requirements for future digital biomarker deployment. Weakness: The manuscript does not describe training–validation–test partition or cross-validation. All analyses appear to be conducted within the same participant sample, meaning the reported AUC values represent in-sample performance rather than true generalization to unseen data. Due to low sample size, I would recommend leave-one-out approach.

- *Thank you for your comment. As recommended, given the available sample size, we implemented leave-one-out cross-validation. All results for the sensitivity analysis are now based on binary logistic regression with leave-one-out cross-validation.*

3. Clinical Trial Sample-Size Estimation: The inclusion of a sample-size analysis for hypothetical neuroprotective trials is conceptually interesting and underscores the translational potential of the proposed linguistic biomarkers. By estimating the number of participants required to detect a 50 % treatment effect, the authors attempt to demonstrate how these LLM-derived metrics could serve as quantitative trial endpoints.
However, this analysis assumes that the language-based biomarkers are already validated, standardized, and generalizable—conditions not yet met in this early proof-of-concept study. The sample-size estimates are derived from a small, single-center cohort and therefore reflect exploratory projections rather than definitive design parameters.
The authors should (1) clearly state that the power analysis is illustrative, not prescriptive for clinical trial planning, and (2) discuss the uncertainty of these estimates given the limited sample size and absence of external validation. Clarifying this point will prevent over-interpretation of the biomarker's readiness for clinical application while still highlighting its potential utility.

- *We agree and have emphasized that this is a proof-of-concept study, and the sample size estimates should be interpreted accordingly. The term "proof-of-concept" has been used consistently throughout the manuscript. In the Methods section under Statistical analysis, we have clarified that the sample size estimation is illustrative, intended to demonstrate the feasibility of linguistic biomarkers rather than to provide prescriptive guidance for clinical trial design. This clarification is also reflected in the Discussion section.*

Minor Comments:

1- GPT-2 vs multi-lingual GPT-2: GPT-2 requires clarification.

- *We clarified that the analyses were conducted using the original GPT-2, including a citation to the paper introducing the model. We did not use any task-specific fine-tuning or adaptation to Czech, as this choice was deliberate to ensure full reproducibility. We have also revised the Methods section introducing GPT-2 to clearly specify which model was used and expanded the description to explicitly note that byte-level tokenization allows the model to process all text, including Czech, without unknown tokens, ensuring that log-likelihoods are well-defined and comparable within our dataset.*

2- Clarify whether the LLM models were run in "frozen-weight" inference mode or fine-tuned on a subset of phone-call transcripts.

- *All models were run in 'frozen-weight' inference mode without further fine-*tuning. *This information has been added to Materials and methods section in the end of Lingustic analysis.*

3- Specify whether embeddings were normalized or standardized across participants before statistical analysis.

- *Features were not standardized prior to any statistical analysis to enhance reproducibility. This clarification has been included in the Statistical Analysis section. Standardization was applied only when constructing the linguistic index, defined as the mean of z-standardized features (added during the review process).*

4- Provide descriptive statistics (mean ± SD) for each linguistic metric at baseline and follow-up in the main text or supplementary tables.

- *We added descriptive statistics to the Supplementary Material (Table S3), reporting means and standard deviations at baseline, 1-year, and 2-year follow-up.*

5- The term "sentence probability" may be unfamiliar to many readers; so include a short mathematical definition (e.g., log-likelihood under GPT-2 language model). Please do this for the three remaining variables.

- *Thank you for your suggestion. We revised Figure 1B to include the mathematical definitions and computational principles of each linguistic feature, and moved the overall description with pathophysiological significance to the Materials and Methods section under Linguistic Analysis.*

6- Report the number of calls and total speech duration per group at baseline versus follow-up in a summary table.

- *We now report these numbers in Supplementary Material under Table S2.*

7- Clarify whether calls from the same participant were kept together within folds if cross-validation is later added.

- *Yes, this was ensured. We have clarified that cross-validation was performed using a leave-one-subject-out approach, so that all data from the test participant are completely unseen during each fold.*

8- The phrase "adapted on Czech data" (Methods, line 16) should cite the dataset used for adaptation. (You may clarify the adjective adopted).

- *We added the citation. The dataset is described in the original paper, which introduces RetroMAE-small, developed by Seznam.cz.*

9- Discussion: add Subheadings to make it easier for the readers to follow the flow of the discussion.

- *Each paragraph in the Discussion section now includes its subheading.*

10- Discussion: emphasize that this is a proof-of-concept pending external replication.

- The phrase 'proof-of-concept' has been added in multiple places throughout the Discussion and further emphasized, as noted above, in relation to the sample size estimation.

11- The Methods section came after the Discussion in the manuscript, so the proper order of sections needs to be established.

- *Thank you for this comment. We have reorganized the sections so that the Methods now follows the Introduction.*

12- Table 1: the duration of iRBD and PD should be included.

- *We believe this information was missed by reviewer and is already presented in the table under "Self-reported symptom duration" and "Disease duration".*

13- Page 9, 10 lines from the bottom of the page: word reversal is needed to change to "not sensitive enough".

- *We changed the phrase accordingly.*

Reviewer #3:
The authors claim the process "requires no patient effort or clinical supervision". This is likely not accurate. The patients are still required to provide data. There is very strong evidence supporting the role of appropriate quality control to data collection and there are large swathes of unsupervised data that yield problematic data when it comes to analysis (noise, inaccurate production, wrong speaker).

- *We agree that in the current approach patients still need to confirm that their recordings can be sent for analysis. We have reformulated the text to emphasize that our approach minimizes the effort required from both patients and clinicians compared with standard clinical measures.*
- *We have expanded the Materials and Methods section under 'Smartphone Phone Call Assessment' and 'Speech Transcription' to provide additional details on the algorithms and processes applied during recording collection and processing. These include detection of excessive noise, identification of the correct speaker, and other mechanisms that ensure only high-quality recordings are analyzed. A full description of the application and algorithms has been previously published (see https://bmjopen.bmj.com/content/12/6/e059871).*

The longitudinal dataset is a great asset, even in this modest sample size.

- *Thank you.*

We really need much more information on the LLM methods. LLMs are a moveable feast. How were each of the methods identified and validated. how was their veracity explained? can you justify their relavance with regard to the clinical phenotype of the chosen population?

- *We have expanded the Linguistic Analysis chapter to introduce the criteria guiding the selection of approaches. These criteria included open-source availability, full local executability, modest computational requirements, and reproducibility. All models were*

evaluated in a frozen-weight inference regime without any fine-tuning to ensure full reproducibility. We emphasize throughout the manuscript that this is a proof-of-concept study; to our knowledge, no prior studies have applied a similar approach to linguistic analysis in neurodegenerative disorders. These findings need to be confirmed in future studies. The relevance to clinical phenotypes is discussed in the Discussion section, in the third paragraph "Linguistic abnormalities and their pathophysiology."

GPT-2 is quite old relative to existing models. can the team use more recent versions?

- *We agree that GPT-2 is a relatively old compared to the newer models However, using newer versions would not fullfill our stated criteria for LLM selection in this proof-of-concept study. We chose GPT-2 because it is fully adequate for our specific task of extracting sentence probabilities (log-likelihoods). Our study does not require state-of-the-art generation or complex reasoning; we only need stable and reliable probability estimates. GPT-2 is fully open-source, enhances transparency, a key consideration in clinical biomarker research. It's lightweight and can run on modest hardware, ensuring that our approach is reproducible and accessible to other researchers. While future studies could explore and compare the efficiency of newer models for similar tasks, our study demonstrates that even this base, older model can extract feature that meaningfully track disease progression.*

can you talk to the quality of the transcription, accuracy of the ASR and how this was verified?

- *Due to privacy policy, we should not manually read or transcribe the phone call recordings, and therefore we did not calculated exact ASR accuracy using these voice calls. However, our previous study indicate that linguistic analysis is robust toward potential transcription error. The* study *(10.1002/mds.29140) compared linguistic features extracted based on automated versus manual transcripts in iRBD and Parkinson's disease, demonstrating high correlation between features (r>0.9) even when the word error rate was up to 18.7%. Nevertheless, errors arising from dysarthric speech or limitations of automatic speech recognition system could still affect the analysis, and we have acknowledged this in the Discussion as a study limitations.*

allowing the participants to review and delete recordings is interesting.

- *Thank you for your comment.*

Can the veracity of this meethod "A speaker recognition system verified that only the participant's voice was present" be confirmed?

- *We expanded the Smartphone phone call assessment section to provide additional details on the algorithms and methods used in this task. A detailed description of the data acquisition process and the specific algorithms has been previously published (see https://doi.org/10.1136/bmjopen-2021-059871). The speaker recognition system is designed to detect the participants. If a participant is not detected—due to low audio quality, low signal-to-noise ratio, or because the participant is not the caller—the audio segment is excluded from further analysis. This step also functions as a quality control check. Additionally, a signal-to-noise estimator is applied during the validation phase, where segments with instantaneous signal-to-noise ratios below a preselected threshold are discarded. We also employed the pyannote voice activity detector to identify segments of participant speech, removing audio in which participants produced fewer than five seconds of speech. The system relies on well-established algorithms specifically*

developed for these tasks. We believe this approach ensures that only high-quality data from the correct speaker are analyzed.

Can the versacity of these measures be explained? "we applied an algorithm to detect and remove such artifacts."

- *We have added a more detailed description of the algorithm developed to limit hallucinations in the Materials and methods section under Speech transcription. The algorithm identifies and removes phrases of at least three words that are repeated consecutively four or more times. These parameters were established and experimentally validated using an internal dataset involving participants with iRBD and PD, where similar transcription artifacts were observed. We believe that the algorithm is now clearly described in the manuscript, and its principles are transparent, enabling full reproducibility.*

The group appear to have called speech and language a biomarker, please consider guidance provided by the NIH and FDA where they state "A biomarker is not a measure of how an individual feels, functions, or survives." please see this website for guidance https://www.ncbi.nlm.nih.gov/books/NBK338448/

- *Thank you for your comment. The guideline clarifies that a biomarker is not a measure of how an individual feels, functions, or survives (e.g., disability scales, symptom questionnaires, or pain ratings). Biomarkers are objective and quantifiable indicators of biological or pathological processes. Speech and language can indeed serve as biomarkers if they are measured objectively, quantified, and reflect underlying biological processes, without relying on subjective symptom reporting. Our linguistic analysis meets all these criteria, and therefore we believe it provides a valid language-based biomarker.*

Reviewer #4:

Thank you for asking me to review this innovative application of LLM in identifying subtypes of language characteristics of patients with RBD, versus those with PD versus controls. The innovative part of the study is the use of smartphones to capture "spontaneous" language and the estimation of the sample size needed (78 patients) for a 2-year neuroprotective trial targeting 50% drug efficacy.

The authors aimed to evaluate whether linguistic features (sentence coherence, semantic diversity and sentence probability) derived from real-world phone calls can serve as a cognitive progressions biomarker in patients with iRBD.

- *Thank you for your positive comments.*

I have only minor points:

1. the link between the linguistic and the cognitive biomarkers is not as yet fully established. Can the authors refrain from using the terms interchangeably (as in the abstract).

- *We agree with this distinction. We have revised the text to ensure we refer to linguistic or language when discussing our direct measurements, and cognition only when discussing the underlying process the features aim to reflect.*

2. Do they have data correlating the linguistic with the acoustic features? the point they make in the discussion about the link between motor speech and linguistic complexity is a valid and logical one.

- *As this study primarily focused on linguistic aspects of speech, acoustic features were not analyzed at this stage. However, based on our findings, both the MDS-UPDRS III total*

score and its speech item declined over time, but they were not associated with changes in linguistic features. This dissociation suggests that the observed linguistic alterations predominantly reflect non-motor language processes rather than motor speech impairment. Thus, future studies may benefit from combining our linguistic approach with motor speech measures to further enhance predictive modeling. We have now addressed this point in the Discussion section.

3. Are there any other insights from their clinical data that could show a larger pattern in the patients who decline in the linguistic domain?

- *Thank you for this insight. We have thoroughly checked all clinical data available with this purpose in mind. Specifically, during the 2-year follow-up period, two iRBD patients developed mild cognitive impairment (MCI). We conducted an additional analysis, dividing the iRBD group into those without MCI (19 patients) and those with MCI (2 patients). We found that iRBD patients with MCI exhibited a significantly greater decline in semantic-syntactic diversity sentence probability, and linguistic index (mean across all linguistic features) compared to those without MCI. In the rest of the linguistic features, even though not signiticant, the decline was also more profound in iRBD with MCI. Although this observation is based on only two participants, it suggests that the emergence of MCI may be associated with accelerated deterioration in language function, highlighting the potential sensitivity of our linguistic biomarkers to early cognitive changes in iRBD. The analysis was included in the Results and is presented in Figure S1.*

Thank you again for allowing me to review this very well written and methodologically innovative paper.

- *Thank you for your careful review of our manuscript.*
