# Supplementary material for: Large language model analysis of real-world phone calls reveals prodromal and progressive biomarker of parkinsonism: A two-year proof-of-concept study
Source: PLOS Digit Health. 2026 Jul 9;5(7):e0001458. doi: 10.1371/journal.pdig.0001458 (PMC13349142; doi:10.1371/journal.pdig.0001458)
Supplement: Attachment — Submitted filename: ResponseToReviewers_R2.pdf [file pdig.0001458.s012.pdf]

Specific responses to reviewers' comments:

Reviewer #1:
The authors have properly addressed all the comments, and the manuscript looks much improved. The revisions to the Methods and Discussion sections have enhanced the clarity, transparency, and interpretability of the work. Overall, the manuscript is suitable for publication.

- *Thank you for your positive feedback.*

I believe there is one remaining typographical error in Statistical analysis section: “These estimates are intended to demonstrate the feasibility linguistic biomarkers and...” should read “the feasibility of linguistic biomarkers”.

- *We corrected the typographical error.*

Reviewer #2:
The revised manuscript addressed all our reviewer comments. Thank you.

- *Thank you for your positive comment.*

Reviewer #5:
This is a revised manuscript (R1) of a two-year longitudinal study investigating the use of Large Language Models (LLMs) to analyze diverse linguistic features from naturalistic phone calls in patients with isolated Rapid Eye Movement Sleep Behavior Disorder (iRBD), Parkinson’s Disease (PD), and healthy controls. The authors collected over 34,000 phone calls and utilized an automated pipeline involving speaker diarization, automated speech recognition (Whisper), and feature extraction via sentence embedding models (RetroMAE-small) and autoregressive models (GPT-2).

The study aims to validate a passive, remote monitoring system that can detect prodromal neurodegeneration. The revised manuscript includes sensitivity analyses for gender (addressing the male-skewed sample), leave-one-out cross-validation (LOOCV), and clarified methodologies regarding hallucination removal in ASR transcripts.

Main Claims
1. A fully automated pipeline can capture and analyze real-world phone conversations without active patient effort, offering a scalable digital biomarker solution.
2. The use of the "sentence probability" linguistic marker could reduce the required sample size for a neuroprotective clinical trial to approximately 78 iRBD participants per arm (targeting 50% drug efficacy), significantly lower than current standards.
3. Patients with iRBD showed a more profound linguistic decline compared to early-stage PD patients, potentially due to the older age of onset or the specific phenotype associated with prodromal Lewy Body Dementia.
4. LLM-derived features (specifically sentence coherence, semantic-syntactic diversity, and sentence probability) detected significant longitudinal decline in iRBD patients over two years, whereas standard cognitive scales (MoCA) did not.

Key Strengths
1. The collection of >34,000 calls in a naturalistic setting is a significant engineering and data collection achievement. This overcomes the "white coat effect" and practice effects seen in

clinic-based active tasks.
2. The shift to leave-one-subject-out cross-validation (LOOCV) for the classification analysis, as requested by Reviewer 2, significantly improves the reliability of the reported AUC values (0.82 for iRBD detection) and reduces the risk of overfitting in this small-N cohort.
3. The authors have implemented a logical and modern processing pipeline (Figure 1). The integration of Voice Activity Detection (pyannote), ASR (Whisper large-v3), and hallucination cleaning algorithms demonstrates a strong understanding of the failure modes in processing spontaneous speech.
4. The authors adequately addressed Reviewer 1's concern regarding the male-dominated sample (95% male). The addition of a sensitivity analysis restricted to males confirmed that the results were not driven by gender imbalance or outliers.

Key Weaknesses
1. While the authors reference a previous study showing high correlation between manual and automated features , the inability to manually verify transcripts due to privacy concerns remains a limitation. Whisper is known to hallucinate, particularly with dysarthric speech or silence. The authors added a heuristic algorithm (removing phrases repeated 4+ times), but subtle hallucinations that alter semantic coherence might still persist and influence the "Sentence Coherence" metric.
2. The use of GPT-2 (released 2019) is justified by the authors for reproducibility and low compute requirements. However, as noted by Reviewer 3, this is an aging architecture. While adequate for calculating log-likelihoods (sentence probability), newer open-weights models (e.g., smaller Llama or Gemma variants) might offer better tokenization and probability estimation for complex sentence structures without significantly higher compute costs.
3. The study utilizes RetroMAE-small, a model adapted for Czech data. While this is excellent for the specific cohort, it raises questions about the immediate transferability of the specific "Semantic-Syntactic Diversity" thresholds to English or other languages without similar high-quality, language-specific embedding models.

- *Thank you for your thorough evaluation of our manuscript. We believe that the key strengths of the study are clearly demonstrated and that its limitations are appropriately justified and discussed in the current version.*

Minor points
1. Figure 1 is highly detailed and effectively illustrates the pipeline. However, it is also extremely complex and somewhat intimidating. In earlier rounds of review, I may have suggested to break this figure up or create sub-panels to better guide the readers attention through the content in an orderly way but at this stage, that suggestion might be more annoying than helpful.

- *We agree that Figure 1 is a complex illustration. To improve clarity, we introduced colored panels to better distinguish healthy and impaired speech examples and added more descriptive captions to guide the reader through the workflow. We believe that these revisions improve readability and overall accessibility of the figure.*

2. The analysis showing that iRBD patients with MCI had steeper linguistic decline is based on a very small sub-sample (n=2). While interesting, the authors properly caveat this, but authors should not overstate this specific finding in the conclusion.

- *We agree. In the Results sections, we stressed out that i tis an exploratory analysis, that have been done only of two iRBD patients, before presenting the results. Furthermore, in the Discussion section, the commenting of these findings where modified and expanded to not overestimate conclusions of these findings. Particularly, the paragraph commenting on these findings have been modified to:*
  "Indeed, the two iRBD participants who developed MCI exhibited a steeper linguistic decline and may indicate a potential association between linguistic decline and emerging cognitive deterioration earlier than conventional screening tools. However, this finding should be interpreted with caution given the very small sub-sample."

3. The discussion mentions future on-device processing. Authors should briefly mention the current latency or processing cost per participant to contextualize the engineering feasibility of scaling this to the n=78 trial size proposed.

- *Thank you for this valuable suggestions. We measured the computational speed of analysis using device we used in this study and reported which part of the analysis is the most computationally costly. There results are reported in the Results section under Participants subsection and further discussed in Discussion section in the paragraph with subtitle "Advantages and impact of passive monitoring in naturalistic environment".*

Overall recommendation:
Accept with minor revisions (points 2 and 3 in Minor points)

- *We thank the reviewer for the constructive comments and valuable suggestions that have helped improve the manuscript.*
