# Supplementary material for: Large language model analysis of real-world phone calls reveals prodromal and progressive biomarker of parkinsonism: A two-year proof-of-concept study
Source: PLOS Digit Health. 2026 Jul 9;5(7):e0001458. doi: 10.1371/journal.pdig.0001458 (PMC13349142; doi:10.1371/journal.pdig.0001458)
Supplement: Peer review history

# Peer review: Decision Letter 0

**DOI:** 10.1371/journal.pdig.0001458.r001

**Authors:** Yuzhe Yang

24 Nov 2025

PDIG-D-25-00765

Large language model analysis of real-world phone calls reveals prodromal and progressive biomarker of parkinsonism: a two-year study

PLOS Digital Health

Dear Dr. Rusz,

Thank you for submitting your manuscript to PLOS Digital Health. After careful consideration, we feel that it has merit but does not fully meet PLOS Digital Health's publication criteria as it currently stands. Therefore, we invite you to submit a revised version of the manuscript that addresses the points raised during the review process.

Please submit your revised manuscript within 60 days Jan 23 2026 11:59PM. If you will need more time than this to complete your revisions, please reply to this message or contact the journal office at digitalhealth@plos.org. When you're ready to submit your revision, log on to https://www.editorialmanager.com/pdig/ and select the 'Submissions Needing Revision' folder to locate your manuscript file.

Please include the following items when submitting your revised manuscript:

* A rebuttal letter that responds to each point raised by the editor and reviewer(s). You should upload this letter as a separate file labeled 'Response to Reviewers'. This file does not need to include responses to any formatting updates and technical items listed in the 'Journal Requirements' section below.

* A marked-up copy of your manuscript that highlights changes made to the original version. You should upload this as a separate file labeled 'Revised Manuscript with Track Changes'.

* An unmarked version of your revised paper without tracked changes. You should upload this as a separate file labeled 'Manuscript'.

If you would like to make changes to your financial disclosure, competing interests statement, or data availability statement, please make these updates within the submission form at the time of resubmission. Guidelines for resubmitting your figure files are available below the reviewer comments at the end of this letter.

We look forward to receiving your revised manuscript.

Kind regards,

Miguel E Rentería, PhD

Academic Editor

PLOS Digital Health

Yuzhe Yang

Section Editor

PLOS Digital Health

Leo Anthony Celi

Editor-in-Chief

PLOS Digital Health

orcid.org/0000-0001-6712-6626

**Journal Requirements:**

1. Please provide an Author Summary. This should appear in your manuscript between the Abstract (if applicable) and the Introduction, and should be 150–200 words long. The aim should be to make your findings accessible to a wide audience that includes both scientists and non-scientists. Sample summaries can be found on our website under Submission Guidelines: [LINK]

https://journals.plos.org/digitalhealth/s/submission-guidelines#loc-parts-of-a-submission

If the reviewer comments include a recommendation to cite specific previously published works, please review and evaluate these publications to determine whether they are relevant and should be cited. There is no requirement to cite these works unless the editor has indicated otherwise.

**Additional Editor Comments (if provided):**

[Note: HTML markup is below. Please do not edit.]

**Reviewers' Comments:**

Reviewer's Responses to Questions

**Comments to the Author**

1. Does this manuscript meet PLOS Digital Health’s publication criteria? Is the manuscript technically sound, and do the data support the conclusions? The manuscript must describe methodologically and ethically rigorous research with conclusions that are appropriately drawn based on the data presented.

Reviewer #1: Partly

Reviewer #2: Yes

Reviewer #3: Partly

Reviewer #4: Yes

**********

2. Has the statistical analysis been performed appropriately and rigorously?

Reviewer #1: Yes

Reviewer #2: No

Reviewer #3: Yes

Reviewer #4: Yes

**********

3. Have the authors made all data underlying the findings in their manuscript fully available (please refer to the Data Availability Statement at the start of the manuscript PDF file)?

The PLOS Data policy requires authors to make all data underlying the findings described in their manuscript fully available without restriction, with rare exception. The data should be provided as part of the manuscript or its supporting information, or deposited to a public repository. For example, in addition to summary statistics, the data points behind means, medians and variance measures should be available. If there are restrictions on publicly sharing data—e.g. participant privacy or use of data from a third party—those must be specified.

Reviewer #1: Yes

Reviewer #2: Yes

Reviewer #3: Yes

Reviewer #4: Yes

**********

4. Is the manuscript presented in an intelligible fashion and written in standard English?

PLOS Digital Health does not copyedit accepted manuscripts, so the language in submitted articles must be clear, correct, and unambiguous. Any typographical or grammatical errors should be corrected at revision, so please note any specific errors here.

Reviewer #1: Yes

Reviewer #2: Yes

Reviewer #3: Yes

Reviewer #4: Yes

**********

5. Review Comments to the Author

Please use the space provided to explain your answers to the questions above. You may also include additional comments for the author, including concerns about dual publication, research ethics, or publication ethics. (Please upload your review as an attachment if it exceeds 20,000 characters)

Reviewer #1: This is an interesting and timely study that addresses the unmet need for early and sensitive biomarkers of synucleinopathies, a topic of substantial relevance to the field of neurodegenerative diseases. The use of LLM-based analysis of real-world telephone speech, combined with a longitudinal and remote design, represents a novel and scalable approach to disease monitoring. The manuscript is clearly written and well structured; however, several aspects require clarification and further substantiation.

1. The study sample is predominantly males, with only one female participant in each subgroup, which is not currently discussed or addressed in the manuscript.

a. Sex differences are known to influence both Parkinsonian manifestations and language characteristics. The inclusion of a single female participant per group introduces the potential for outlier effects, where a small number of atypical datapoints (due to sex-based differences) could disproportionately influence estimates or variance. A sensitivity analysis restricted to male participants would help assess the robustness of the reported findings.

b. This extreme sex imbalance of sample composition limits the interpretability and generalizability of the findings to female patients, as. The authors should explicitly acknowledge this limitation in the Methods, Results, and Discussion sections, and consider mentioning it briefly in the Abstract.

2. This study used the Whisper large-v3 model for audio transcription.

a. While Whisper is highly accurate for general ASR, hallucination is a common issue due to its extensive training on sources such as YouTube. The model can fabricate words or sentences (e.g., “thanks for watching,” “thank you”, etc.) that were not actually spoken, particularly during periods of silence, and it can also repeat phrases or get stuck in loops. The authors mention applying an algorithm to detect and remove these artifacts, but no details were provided regarding how this was performed (automatic or manual, using existing tools or a self-developed method) and how effectively it removed such errors. This is a crucial point, as the linguistic measures of interest in this study are sentence-based.

b. The study used a custom-developed application that recorded only the participant’s speech during phone calls. Were trailing silences or long within-utterance pauses removed during audio processing of these recordings? This is important, as prolonged silences could affect Whisper’s transcription accuracy and potentially introduce artifacts, as noted above.

c. Transcription errors, especially in the context of dysarthric speech, and autocorrection could also bias the results. The authors should clarify whether these issues were checked and should at minimum acknowledge them as limitations.

3. Could the authors provide more details on audio processing? Were recordings discarded only based on insufficient linguistic content (e.g., calls with <5 seconds of participant speech)? Were all retained recordings of sufficient quality for transcription and feature extraction? Were any recordings excluded for other reasons?

4. The study reported relatively high accuracy; however:

a. Validation or test set evaluation are necessary to assess the model’s generalizability and its applicability to real-world data. Given the study sample size, a conventional train/test split may not be feasible. The authors could consider holding out a subset of phone calls, cross-validation, or, if possible, an external dataset to evaluate the robustness and generalizability of the model.

b. The modelling was performed separately for each linguistic domain, which is appropriate for understanding specific language changes and identifying features most relevant to disease progression. Nonetheless, the authors could also consider a combined modelling approach across domains. This would provide a more comprehensive capture of language alterations and could enhance predictive performance.

c. Although both the MDS-UPDRS III total and speech item declined over time, they showed no correlation with language changes. This suggests that the observed linguistic alterations reflect non-motor processes and that incorporating motor speech features could further enhance the model’s predictive performance. While this may be beyond the scope of the current study, the authors could consider discussing this point, as it may have implications for future predictive modelling that fully leverages speech analysis and biomarkers.

5. Finally, a minor point regarding the statistical tests used to compare demographic and clinical characteristics: several categorical variables have very small cell sizes (<5), making the Chi-squared test inappropriate. Fisher’s exact test should be used instead for these comparisons.

6. A few minor typographical points:

a) In the legend of Figure 2, panel (D) reads “Optimal LLM-based linguistic characteristics identifies via …”; the authors likely meant “identified.”

b) In Figure 3, the “sentence probability” section may contain a typo: “language patters” should probably be “language patterns.”

Reviewer #2: General Comments:

The authors presented an innovative and highly promising approach that leverages large language models (LLMs) to extract linguistic biomarkers (digital bio-markers) from real-world phone calls for monitoring disease progression in isolated REM sleep behavior disorder (iRBD) and Parkinson’s disease (PD). The reported linguistic biomarker could possibly be used in future disease-modifying trials.

The study’s design, using passive, naturalistic phone conversations, is novel and aligns with current trends in developing scalable, low-burden digital health biomarkers.

From the AI/LLM standpoint, the concept and rationale are strong, and the integration of sentence embeddings (RetroMAE-small) and GPT-2–derived linguistic metrics is appropriate for capturing cognitive-linguistic decline.

Major Comments:

1. Due to the low sample size, I would highly recommend using the word “proof-of-concept” in the title & the main body of text, i.e., methods section, and also in the abstract.

2. Strength: The inclusion of a test–retest reliability analysis using ICC is commendable and demonstrates thoughtful attention to the intra-individual stability of the LLM-derived linguistic features. Establishing that 18–21 phone calls are sufficient for consistent measurement is a useful step toward defining the data requirements for future digital biomarker deployment. Weakness: The manuscript does not describe training–validation–test partition or cross-validation. All analyses appear to be conducted within the same participant sample, meaning the reported AUC values represent in-sample performance rather than true generalization to unseen data. Due to low sample size, I would recommend leave-one-out approach.

3. Clinical Trial Sample-Size Estimation: The inclusion of a sample-size analysis for hypothetical neuroprotective trials is conceptually interesting and underscores the translational potential of the proposed linguistic biomarkers. By estimating the number of participants required to detect a 50 % treatment effect, the authors attempt to demonstrate how these LLM-derived metrics could serve as quantitative trial endpoints.

However, this analysis assumes that the language-based biomarkers are already validated, standardized, and generalizable—conditions not yet met in this early proof-of-concept study. The sample-size estimates are derived from a small, single-center cohort and therefore reflect exploratory projections rather than definitive design parameters.

The authors should (1) clearly state that the power analysis is illustrative, not prescriptive for clinical trial planning, and (2) discuss the uncertainty of these estimates given the limited sample size and absence of external validation. Clarifying this point will prevent over-interpretation of the biomarker’s readiness for clinical application while still highlighting its potential utility.

Minor Comments:

1- GPT-2 vs multi-lingual GPT-2: GPT-2 requires clarification.

2- Clarify whether the LLM models were run in “frozen-weight” inference mode or fine-tuned on a subset of phone-call transcripts.

3- Specify whether embeddings were normalized or standardized across participants before statistical analysis.

4- Provide descriptive statistics (mean ± SD) for each linguistic metric at baseline and follow-up in the main text or supplementary tables.

5- The term “sentence probability” may be unfamiliar to many readers; so include a short mathematical definition (e.g., log-likelihood under GPT-2 language model). Please do this for the three remaining variables.

6- Report the number of calls and total speech duration per group at baseline versus follow-up in a summary table.

7- Clarify whether calls from the same participant were kept together within folds if cross-validation is later added.

8- The phrase “adapted on Czech data” (Methods, line 16) should cite the dataset used for adaptation. (You may clarify the adjective adopted).

9- Discussion: add Subheadings to make it easier for the readers to follow the flow of the discussion.

10- Discussion: emphasize that this is a proof-of-concept pending external replication.

11- The Methods section came after the Discussion in the manuscript, so the proper order of sections needs to be established.

12- Table 1: the duration of iRBD and PD should be included.

13- Page 9, 10 lines from the bottom of the page: word reversal is needed to change to “not sensitive enough”.

Reviewer #3: The authors claim the process "requires no patient effort or clinical supervision". This is likely not accurate. The patients are still required to provide data. There is very strong evidence supporting the role of appropriate quality control to data collection and there are large swathes of unsupervised data that yield problematic data when it comes to analysis (noise, inaccurate production, wrong speaker).

The longitudinal dataset is a great asset, even in this modest sample size.

We really need much more information on the LLM methods. LLMs are a moveable feast. How were each of the methods identified and validated. how was their veracity explained? can you justify their relavance with regard to the clinical phenotype of the chosen population?

GPT-2 is quite old relative to existing models. can the team use more recent versions?

can you talk to the quality of the transcription, accuracy of the ASR and how this was verified?

allowing the participants to review and delete recordings is interesting.

Can the veracity of this meethod "A speaker recognition system verified that only the participant’s voice was

present" be confirmed?

Can the versacity of these measures be explained? "we applied an algorithm to detect and remove such artifacts."

The group appear to have called speech and language a biomarker, please consider guidance provided by the NIH and FDA where they state "A biomarker is not a measure of how an individual feels, functions, or survives." please see this website for guidance https://www.ncbi.nlm.nih.gov/books/NBK338448/

Reviewer #4: Thank you for asking me to review this innovative application of LLM in identifying subtypes of language characteristics of patients with RBD, versus those with PD versus controls. The innovative part of the study is the use of smartphones to capture "spontaneous" language and the estimation of the sample size needed (78 patients) for a 2-year neuroprotective trial targeting 50% drug efficacy.

The authors aimed to evaluate whether linguistic features (sentence coherence, semantic diversity and sentence probability) derived from real-world phone calls can serve as a cognitive progressions biomarker in patients with iRBD.

I have only minor points:

1. the link between the linguistic and the cognitive biomarkers is not as yet fully established.

Can the authors refrain from using the terms interchangeably (as in the abstract).

2. Do they have data correlating the linguistic with the acoustic features? the point they make in the discussion about the link between motor speech and linguistic complexity is a valid and logical one.

3. Are there any other insights from their clinical data that could show a larger pattern in the patients who decline in the linguistic domain?

Thank you again for allowing me to review this very well written and methodologically innovative paper.

**********

6. PLOS authors have the option to publish the peer review history of their article (what does this mean?). If published, this will include your full peer review and any attached files.

**Do you want your identity to be public for this peer review?**  If you choose “no”, your identity will remain anonymous but your review may still be made public.

For information about this choice, including consent withdrawal, please see our Privacy Policy.

Reviewer #1: **Yes:** Fangyuan Cao

Reviewer #2: **Yes:** Javad Razjouyan and Carlos H. Schenck

Reviewer #3: No

Reviewer #4: **Yes:** Elina Tripoliti

**********

[NOTE: If reviewer comments were submitted as an attachment file, they will be attached to this email and accessible via the submission site. Please log into your account, locate the manuscript record, and check for the action link "View Attachments". If this link does not appear, there are no attachment files.]

**Figure resubmission:**

While revising your submission, we strongly recommend that you use PLOS’s NAAS tool (https://ngplosjournals.pagemajik.ai/artanalysis) to test your figure files. NAAS can convert your figure files to the TIFF file type and meet basic requirements (such as print size, resolution), or provide you with a report on issues that do not meet our requirements and that NAAS cannot fix.

After uploading your figures to PLOS’s NAAS tool - https://ngplosjournals.pagemajik.ai/artanalysis, NAAS will process the files provided and display the results in the "Uploaded Files" section of the page as the processing is complete. If the uploaded figures meet our requirements (or NAAS is able to fix the files to meet our requirements), the figure will be marked as "fixed" above. If NAAS is unable to fix the files, a red "failed" label will appear above. When NAAS has confirmed that the figure files meet our requirements, please download the file via the download option, and include these NAAS processed figure files when submitting your revised manuscript.

**Reproducibility:**

To enhance the reproducibility of your results, we recommend that authors of applicable studies deposit laboratory protocols in protocols.io, where a protocol can be assigned its own identifier (DOI) such that it can be cited independently in the future. Additionally, PLOS ONE offers an option to publish peer-reviewed clinical study protocols. Read more information on sharing protocols at https://plos.org/protocols?utm_medium=editorial-email&utm_source=authorletters&utm_campaign=protocols

# Author comment: Author response to Decision Letter 1

**DOI:** 10.1371/journal.pdig.0001458.r002

22 Dec 2025

Attachment Submitted filename: ResponseToReviews_R1.pdf

# Peer review: Decision Letter 1

**DOI:** 10.1371/journal.pdig.0001458.r003

**Authors:** Yuzhe Yang

16 Mar 2026

PDIG-D-25-00765R1

Large language model analysis of real-world phone calls reveals prodromal and progressive biomarker of parkinsonism: a two-year proof-of-concept study

PLOS Digital Health

Dear Dr. Rusz,

Thank you for submitting your manuscript to PLOS Digital Health. After careful consideration, we feel that it has merit but does not fully meet PLOS Digital Health's publication criteria as it currently stands. Therefore, we invite you to submit a revised version of the manuscript that addresses the points raised during the review process.

Please submit your revised manuscript by Apr 15 2026 11:59PM. If you will need more time than this to complete your revisions, please reply to this message or contact the journal office at digitalhealth@plos.org. When you're ready to submit your revision, log on to https://www.editorialmanager.com/pdig/ and select the 'Submissions Needing Revision' folder to locate your manuscript file.

Please include the following items when submitting your revised manuscript:

* A letter that responds to each point raised by the editor and reviewer(s). You should upload this letter as a separate file labeled 'Response to Reviewers'. This file does not need to include responses to any formatting updates and technical items listed in the 'Journal Requirements' section below.

* A marked-up copy of your manuscript that highlights changes made to the original version. You should upload this as a separate file labeled 'Revised Manuscript with Track Changes'.

* An unmarked version of your revised paper without tracked changes. You should upload this as a separate file labeled 'Manuscript'.

If you would like to make changes to your financial disclosure, competing interests statement, or data availability statement, please make these updates within the submission form at the time of resubmission. Guidelines for resubmitting your figure files are available below the reviewer comments at the end of this letter.

We look forward to receiving your revised manuscript.

Kind regards,

Miguel E Rentería

Academic Editor

PLOS Digital Health

Yuzhe Yang

Section Editor

PLOS Digital Health

Leo Anthony Celi

Editor-in-Chief

PLOS Digital Health

orcid.org/0000-0001-6712-6626

**Journal Requirements:**

If the reviewer comments include a recommendation to cite specific previously published works, please review and evaluate these publications to determine whether they are relevant and should be cited. There is no requirement to cite these works unless the editor has indicated otherwise.

**Additional Editor Comments (if provided):**

Summary: This is a revised manuscript (R1) of a two-year longitudinal study investigating the use of Large Language Models (LLMs) to analyze diverse linguistic features from naturalistic phone calls in patients with isolated Rapid Eye Movement Sleep Behavior Disorder (iRBD), Parkinson’s Disease (PD), and healthy controls. The authors collected over 34,000 phone calls and utilized an automated pipeline involving speaker diarization, automated speech recognition (Whisper), and feature extraction via sentence embedding models (RetroMAE-small) and autoregressive models (GPT-2).

The study aims to validate a passive, remote monitoring system that can detect prodromal neurodegeneration. The revised manuscript includes sensitivity analyses for gender (addressing the male-skewed sample), leave-one-out cross-validation (LOOCV), and clarified methodologies regarding hallucination removal in ASR transcripts.

Main Claims

1. A fully automated pipeline can capture and analyze real-world phone conversations without active patient effort, offering a scalable digital biomarker solution.

2. The use of the "sentence probability" linguistic marker could reduce the required sample size for a neuroprotective clinical trial to approximately 78 iRBD participants per arm (targeting 50% drug efficacy), significantly lower than current standards.

3. Patients with iRBD showed a more profound linguistic decline compared to early-stage PD patients, potentially due to the older age of onset or the specific phenotype associated with prodromal Lewy Body Dementia.

4. LLM-derived features (specifically sentence coherence, semantic-syntactic diversity, and sentence probability) detected significant longitudinal decline in iRBD patients over two years, whereas standard cognitive scales (MoCA) did not.

Key Strengths

1. The collection of >34,000 calls in a naturalistic setting is a significant engineering and data collection achievement. This overcomes the "white coat effect" and practice effects seen in clinic-based active tasks.

2. The shift to leave-one-subject-out cross-validation (LOOCV) for the classification analysis, as requested by Reviewer 2, significantly improves the reliability of the reported AUC values (0.82 for iRBD detection) and reduces the risk of overfitting in this small-N cohort.

3. The authors have implemented a logical and modern processing pipeline (Figure 1). The integration of Voice Activity Detection (pyannote), ASR (Whisper large-v3), and hallucination cleaning algorithms demonstrates a strong understanding of the failure modes in processing spontaneous speech.

4. The authors adequately addressed Reviewer 1's concern regarding the male-dominated sample (95% male). The addition of a sensitivity analysis restricted to males confirmed that the results were not driven by gender imbalance or outliers.

Key Weaknesses

1. While the authors reference a previous study showing high correlation between manual and automated features , the inability to manually verify transcripts due to privacy concerns remains a limitation. Whisper is known to hallucinate, particularly with dysarthric speech or silence. The authors added a heuristic algorithm (removing phrases repeated 4+ times), but subtle hallucinations that alter semantic coherence might still persist and influence the "Sentence Coherence" metric.

2. The use of GPT-2 (released 2019) is justified by the authors for reproducibility and low compute requirements. However, as noted by Reviewer 3, this is an aging architecture. While adequate for calculating log-likelihoods (sentence probability), newer open-weights models (e.g., smaller Llama or Gemma variants) might offer better tokenization and probability estimation for complex sentence structures without significantly higher compute costs.

3. The study utilizes RetroMAE-small, a model adapted for Czech data. While this is excellent for the specific cohort, it raises questions about the immediate transferability of the specific "Semantic-Syntactic Diversity" thresholds to English or other languages without similar high-quality, language-specific embedding models.

Minor points

1. Figure 1 is highly detailed and effectively illustrates the pipeline. However, it is also extremely complex and somewhat intimidating. In earlier rounds of review, I may have suggested to break this figure up or create sub-panels to better guide the readers attention through the content in an orderly way but at this stage, that suggestion might be more annoying than helpful.

2. The analysis showing that iRBD patients with MCI had steeper linguistic decline is based on a very small sub-sample (n=2). While interesting, the authors properly caveat this, but authors should not overstate this specific finding in the conclusion.

3. The discussion mentions future on-device processing. Authors should briefly mention the current latency or processing cost per participant to contextualize the engineering feasibility of scaling this to the n=78 trial size proposed.

[Note: HTML markup is below. Please do not edit.]

**Reviewers' Comments:**

Reviewer's Responses to Questions

**Comments to the Author**

1. If the authors have adequately addressed your comments raised in a previous round of review and you feel that this manuscript is now acceptable for publication, you may indicate that here to bypass the “Comments to the Author” section, enter your conflict of interest statement in the “Confidential to Editor” section, and submit your "Accept" recommendation.

Reviewer #1: All comments have been addressed

Reviewer #2: All comments have been addressed

Reviewer #5: (No Response)

**********

2. Does this manuscript meet PLOS Digital Health’s publication criteria? Is the manuscript technically sound, and do the data support the conclusions? The manuscript must describe methodologically and ethically rigorous research with conclusions that are appropriately drawn based on the data presented.

Reviewer #1: Yes

Reviewer #2: Yes

Reviewer #5: Yes

**********

3. Has the statistical analysis been performed appropriately and rigorously?

Reviewer #1: Yes

Reviewer #2: Yes

Reviewer #5: Yes

**********

4. Have the authors made all data underlying the findings in their manuscript fully available (please refer to the Data Availability Statement at the start of the manuscript PDF file)?

The PLOS Data policy requires authors to make all data underlying the findings described in their manuscript fully available without restriction, with rare exception. The data should be provided as part of the manuscript or its supporting information, or deposited to a public repository. For example, in addition to summary statistics, the data points behind means, medians and variance measures should be available. If there are restrictions on publicly sharing data—e.g. participant privacy or use of data from a third party—those must be specified.

Reviewer #1: Yes

Reviewer #2: Yes

Reviewer #5: Yes

**********

5. Is the manuscript presented in an intelligible fashion and written in standard English?

PLOS Digital Health does not copyedit accepted manuscripts, so the language in submitted articles must be clear, correct, and unambiguous. Any typographical or grammatical errors should be corrected at revision, so please note any specific errors here.

Reviewer #1: Yes

Reviewer #2: Yes

Reviewer #5: Yes

**********

6. Review Comments to the Author

Please use the space provided to explain your answers to the questions above. You may also include additional comments for the author, including concerns about dual publication, research ethics, or publication ethics. (Please upload your review as an attachment if it exceeds 20,000 characters)

**Reviewer #1:** The authors have properly addressed all the comments, and the manuscript looks much improved. The revisions to the Methods and Discussion sections have enhanced the clarity, transparency, and interpretability of the work. Overall, the manuscript is suitable for publication.

I believe there is one remaining typographical error in Statistical analysis section: “These estimates are intended to

demonstrate the feasibility linguistic biomarkers and...” should read “the feasibility of linguistic biomarkers”.

**Reviewer #2:**  The revised manuscript addressed all our reviewer comments. Thank you.

**Reviewer #5:** Summary

This is a revised manuscript (R1) of a two-year longitudinal study investigating the use of Large Language Models (LLMs) to analyze diverse linguistic features from naturalistic phone calls in patients with isolated Rapid Eye Movement Sleep Behavior Disorder (iRBD), Parkinson’s Disease (PD), and healthy controls. The authors collected over 34,000 phone calls and utilized an automated pipeline involving speaker diarization, automated speech recognition (Whisper), and feature extraction via sentence embedding models (RetroMAE-small) and autoregressive models (GPT-2).

The study aims to validate a passive, remote monitoring system that can detect prodromal neurodegeneration. The revised manuscript includes sensitivity analyses for gender (addressing the male-skewed sample), leave-one-out cross-validation (LOOCV), and clarified methodologies regarding hallucination removal in ASR transcripts.

Main Claims

1. A fully automated pipeline can capture and analyze real-world phone conversations without active patient effort, offering a scalable digital biomarker solution.

2. The use of the "sentence probability" linguistic marker could reduce the required sample size for a neuroprotective clinical trial to approximately 78 iRBD participants per arm (targeting 50% drug efficacy), significantly lower than current standards.

3. Patients with iRBD showed a more profound linguistic decline compared to early-stage PD patients, potentially due to the older age of onset or the specific phenotype associated with prodromal Lewy Body Dementia.

4. LLM-derived features (specifically sentence coherence, semantic-syntactic diversity, and sentence probability) detected significant longitudinal decline in iRBD patients over two years, whereas standard cognitive scales (MoCA) did not.

Key Strengths

1. The collection of >34,000 calls in a naturalistic setting is a significant engineering and data collection achievement. This overcomes the "white coat effect" and practice effects seen in clinic-based active tasks.

2. The shift to leave-one-subject-out cross-validation (LOOCV) for the classification analysis, as requested by Reviewer 2, significantly improves the reliability of the reported AUC values (0.82 for iRBD detection) and reduces the risk of overfitting in this small-N cohort.

3. The authors have implemented a logical and modern processing pipeline (Figure 1). The integration of Voice Activity Detection (pyannote), ASR (Whisper large-v3), and hallucination cleaning algorithms demonstrates a strong understanding of the failure modes in processing spontaneous speech.

4. The authors adequately addressed Reviewer 1's concern regarding the male-dominated sample (95% male). The addition of a sensitivity analysis restricted to males confirmed that the results were not driven by gender imbalance or outliers.

Key Weaknesses

1. While the authors reference a previous study showing high correlation between manual and automated features , the inability to manually verify transcripts due to privacy concerns remains a limitation. Whisper is known to hallucinate, particularly with dysarthric speech or silence. The authors added a heuristic algorithm (removing phrases repeated 4+ times), but subtle hallucinations that alter semantic coherence might still persist and influence the "Sentence Coherence" metric.

2. The use of GPT-2 (released 2019) is justified by the authors for reproducibility and low compute requirements. However, as noted by Reviewer 3, this is an aging architecture. While adequate for calculating log-likelihoods (sentence probability), newer open-weights models (e.g., smaller Llama or Gemma variants) might offer better tokenization and probability estimation for complex sentence structures without significantly higher compute costs.

3. The study utilizes RetroMAE-small, a model adapted for Czech data. While this is excellent for the specific cohort, it raises questions about the immediate transferability of the specific "Semantic-Syntactic Diversity" thresholds to English or other languages without similar high-quality, language-specific embedding models.

Minor points

1. Figure 1 is highly detailed and effectively illustrates the pipeline. However, it is also extremely complex and somewhat intimidating. In earlier rounds of review, I may have suggested to break this figure up or create sub-panels to better guide the readers attention through the content in an orderly way but at this stage, that suggestion might be more annoying than helpful.

2. The analysis showing that iRBD patients with MCI had steeper linguistic decline is based on a very small sub-sample (n=2). While interesting, the authors properly caveat this, but authors should not overstate this specific finding in the conclusion.

3. The discussion mentions future on-device processing. Authors should briefly mention the current latency or processing cost per participant to contextualize the engineering feasibility of scaling this to the n=78 trial size proposed.

Overall recommendation:

Accept with minor revisions (points 2 and 3 in Minor points)

**********

7. PLOS authors have the option to publish the peer review history of their article (what does this mean?). If published, this will include your full peer review and any attached files.

**Do you want your identity to be public for this peer review?**  If you choose “no”, your identity will remain anonymous but your review may still be made public.

For information about this choice, including consent withdrawal, please see our Privacy Policy.

Reviewer #1: **Yes:** Fangyuan Cao

Reviewer #2: No

Reviewer #5: No

**********

[NOTE: If reviewer comments were submitted as an attachment file, they will be attached to this email and accessible via the submission site. Please log into your account, locate the manuscript record, and check for the action link "View Attachments". If this link does not appear, there are no attachment files.]

**Figure resubmission:**

While revising your submission, we strongly recommend that you use PLOS’s NAAS tool (https://ngplosjournals.pagemajik.ai/artanalysis) to test your figure files. NAAS can convert your figure files to the TIFF file type and meet basic requirements (such as print size, resolution), or provide you with a report on issues that do not meet our requirements and that NAAS cannot fix.

After uploading your figures to PLOS’s NAAS tool - https://ngplosjournals.pagemajik.ai/artanalysis, NAAS will process the files provided and display the results in the "Uploaded Files" section of the page as the processing is complete. If the uploaded figures meet our requirements (or NAAS is able to fix the files to meet our requirements), the figure will be marked as "fixed" above. If NAAS is unable to fix the files, a red "failed" label will appear above. When NAAS has confirmed that the figure files meet our requirements, please download the file via the download option, and include these NAAS processed figure files when submitting your revised manuscript.

**Reproducibility:**

To enhance the reproducibility of your results, we recommend that authors of applicable studies deposit laboratory protocols in protocols.io, where a protocol can be assigned its own identifier (DOI) such that it can be cited independently in the future. Additionally, PLOS ONE offers an option to publish peer-reviewed clinical study protocols. Read more information on sharing protocols at https://plos.org/protocols?utm_medium=editorial-email&utm_source=authorletters&utm_campaign=protocols

# Author comment: Author response to Decision Letter 2

**DOI:** 10.1371/journal.pdig.0001458.r004

18 Mar 2026

Attachment Submitted filename: ResponseToReviewers_R2.pdf

# Editor report: Decision Letter 2

**DOI:** 10.1371/journal.pdig.0001458.r005

**Authors:** Yuzhe Yang

8 May 2026

Large language model analysis of real-world phone calls reveals prodromal and progressive biomarker of parkinsonism: a two-year proof-of-concept study

PDIG-D-25-00765R2

Dear Dr. Rusz,

We are pleased to inform you that your manuscript 'Large language model analysis of real-world phone calls reveals prodromal and progressive biomarker of parkinsonism: a two-year proof-of-concept study' has been provisionally accepted for publication in PLOS Digital Health.

Before your manuscript can be formally accepted you will need to complete some formatting changes, which you will receive in a follow-up email from a member of our team.

Please note that your manuscript will not be scheduled for publication until you have made the required changes, so a swift response is appreciated.

IMPORTANT: The editorial review process is now complete. PLOS will only permit corrections to spelling, formatting or significant scientific errors from this point onwards. Requests for major changes, or any which affect the scientific understanding of your work, will cause delays to the publication date of your manuscript.

If your institution or institutions have a press office, please notify them about your upcoming paper to help maximize its impact. If they'll be preparing press materials, please inform our press team as soon as possible -- no later than 48 hours after receiving the formal acceptance. Your manuscript will remain under strict press embargo until 2 pm Eastern Time on the date of publication. For more information, please contact digitalhealth@plos.org.

Thank you again for supporting Open Access publishing; we are looking forward to publishing your work in PLOS Digital Health.

Best regards,

Miguel E Rentería, PhD

Academic Editor

PLOS Digital Health

***********************************************************

**Additional Editor Comments (if provided):**

**Reviewer Comments (if any, and for reference):**
